# Coupled intelligent prediction model for medium- to long-term runoff based on teleconnection factors selection and spatial-temporal analysis

**DOI:** 10.1371/journal.pone.0313871

**Published:** 2024-12-12

**Authors:** Jintao Li, Ping Ai, Chuansheng Xiong, Yanhong Song

**Affiliations:** 1 College of Computer Science and Software Engineering, Hohai University, Nanjing, China; 2 College of Hydrology and Water Resources, Hohai University, Nanjing, China; University 20 Aout 1955 skikda, Algeria, ALGERIA

## Abstract

Accurate medium- to long-term runoff forecasting is of great significance for flood control, drought mitigation, comprehensive water resource management, and ecological restoration. However, runoff formation is a complex process influenced by various natural and anthropogenic factors, resulting in nonlinearity, nonstationarity, and long prediction periods, which complicate forecasting efforts. Traditional statistical models, which primarily focus on individual runoff sequences, struggle to integrate multi-source data, limiting their predictive accuracy. This narrow approach overlooks the multifaceted variables influencing runoff, resulting in incomplete and less reliable predictions. To address these challenges, we selected and integrated Random Forest (RF), Support Vector Regression (SVR), and Multilayer Perceptron Regression (MLPR) to develop two coupled intelligent prediction models—RF-SVR and RF-MLPR—due to their complementary strengths. RF effectively removes collinear and redundant information from high-dimensional data, while SVR and MLPR handle nonlinearity and nonstationarity, offering enhanced generalization capabilities. Specifically, MLPR, with its deep learning structure, can extract more complex latent information from data, making it particularly suitable for long-term forecasting. The proposed models were tested in the Yalong River Basin (YLRB), where accurate medium- to long-term runoff forecasts are essential for ecological management, flood control, and optimal water resource allocation. The results demonstrate the following: (1) The impact of atmospheric circulation indices on YLRB runoff exhibits a one-month lag, providing crucial insights for water resource scheduling and flood prevention. (2) The coupled models effectively eliminate collinearity and redundant variables, improving prediction accuracy across all forecast periods. (3) Compared to single baseline models, the coupled models demonstrated significant performance improvements across six evaluation metrics. For instance, the RF-MLPR model achieved a 3.7%–6.5% improvement in the Nash-Sutcliffe efficiency (NSE) metric across four hydrological stations compared to the RF-SVR model. (4) Prediction accuracy decreased with longer forecast periods, with the R^2^ value dropping from 0.8886 for a 1-month forecast to 0.6358 for a 12-month forecast, indicating the increasing challenge of long-term predictions due to greater uncertainty and the accumulation of influencing factors over time. (5) The RF-MLPR model outperformed the RF-SVR model, demonstrating a superior ability to capture the complex, nonlinear relationships inherent in the data. For example, in terms of the R^2^ metric, the RF-MLPR model’s performance at the Jinping hydrological station improved by 6.5% compared to the RF-SVR model. Similarly, at the Lianghekou station, for a one-month lead prediction period, the RF-MLPR model’s R^2^ value was 7.9% higher than that of the RF-SVR model. The significance of this research lies not only in its contribution to improving hydrological prediction accuracy but also in its broader applicability. The proposed coupled prediction models provide practical tools for water resource management, flood control planning, and drought mitigation in regions with similar hydrological characteristics. Furthermore, the framework’s flexibility in parameterization and its ability to integrate multi-source data offer valuable insights for interdisciplinary applications across environmental sciences, meteorology, and climate prediction, making it a globally relevant contribution to addressing water management challenges under changing climatic conditions.

## 1 Introduction

Accurate and reliable medium- to long-term runoff prediction holds significant importance for rational water resource planning and management, enhancing flood control and disaster reduction capabilities, maximizing the comprehensive benefits of reservoirs, and improving the ecological environment [1–4]. Runoff formation is influenced by a multitude of natural and anthropogenic factors, constituting a complex process characterized by nonstationarity and nonlinearity [5]. It is difficult to obtain high prediction accuracy by traditional methods that only consider single sequence data and series methods based on statistics [6, 7], which increasingly cannot meet the development needs of current society and production. Therefore, in the field of hydrological forecasting, research is dedicated to introducing runoff prediction models that exhibit good applicability and can effectively integrate multiple sources of influencing factors to achieve higher accuracy in runoff predictions. This has become a prominent and challenging topic in this research field.

Due to the current lack of reliable meteorological forecasts and precipitation data to support medium- to long-term runoff predictions, the uncertainty of these predictions remains relatively high. In recent years, scholars have incorporated remotely sensed variables into hydrological research [8–10], significantly improving the accuracy of runoff and precipitation forecasts. Studies have demonstrated that teleconnection indices, such as the Pacific Decadal Oscillation (PDO) and the El Niño-Southern Oscillation (ENSO), are closely linked to surface runoff and precipitation across various regions worldwide [11, 12]. For instance, runoff variations in the Pearl River Basin are associated with the Indian Ocean Dipole (IOD), while the winter climate in Europe is strongly influenced by the North Atlantic Oscillation (NAO) and other teleconnection patterns [13, 14]. Similarly, atmospheric teleconnection patterns impact surface temperature anomalies in Western Eurasia through changes in cloud cover and water vapor effects [15]. The interaction between the East Asian winter monsoon and the Australian summer monsoon has been found to promote El Niño events [16], and runoff in Trinity Lake, California, shows significant correlations with multiple climate indices [17].

Moreover, teleconnection patterns have been widely applied to improve the accuracy of forecasts in various hydrological and climate-related fields. Progress has been made in rainfall forecasting [18–20], flood forecasting [21–23], snowmelt prediction [24–28], drought prediction [29], hydroclimate change analysis [30], and long-term extreme temperature change predictions [31]. These studies highlight potential teleconnection patterns between climate factors and water resource variability, providing valuable insights for improving forecast precision and supporting hydrological and climate research.

The introduction of teleconnection index variables such as meteorological elements enriches the multidimensional description of hydrological processes but also leads to a rapid increase in data dimensions. Typically, high-dimensional datasets contain a large amount of collinear and redundant information, making it challenging to achieve good results directly using untreated high-dimensional data for classification or prediction tasks. Traditionally, relevant factors are selected based on the correlation between atmospheric circulation indices and target hydrological sequence data. Then, the selected factors are used for regression analysis of the target sequence variables. However, it should be noted that variables selected solely based on the correlation between a single factor and complex sequence data are not typical and cannot be used for analyzing and explaining comprehensive geophysical sequences. Moreover, non-stationary hydrological sequence data are not suitable for regression analysis using traditional statistical methods [32].

The random forest (RF) algorithm is an effective tool for feature selection in high-dimensional data [33]. It can comprehensively analyze nonlinear and implicit relationships between independent and target variables in multi-source datasets, removing collinear and redundant data to improve model efficiency. As a representative of ensemble learning algorithms, RF has been widely applied in various fields. For instance, it has demonstrated superior performance over other feature selection methods in numerous University of California, Irvine (UCI) datasets, achieving reduced computational costs [34]. Additionally, RF has been employed to identify crucial phenological features of corn and soybeans in the U.S. Corn Belt, revealing spatial-temporal patterns that support detailed classification of important variables [35]. Another study showed that applying RF to nucleotide sequences improved model accuracy by 3% through optimal feature selection [36].

Hydrological processes are highly complex, nonlinear, and nonstationary systems influenced by various intertwined factors. Traditional statistical models, such as autocorrelation, autoregression, ARMA, and ARIMA, struggle to incorporate multi-source data, resulting in low predictive accuracy. In contrast, data-driven models like Support Vector Regression (SVR) [37] and Multilayer Perceptron Regression (MLPR) [38–40] have garnered attention for their ability to explore complex relationships between observational data and prediction targets. These models demonstrate robust fitting and generalization capabilities when handling nonstationary, nonlinear, and high-dimensional data.

For example, the SVR model has been successfully applied to high-accuracy predictions of soil pollution conditions [41]. Moreover, its integration with the Grey Wolf Optimization (GWO) algorithm has yielded optimal results in landslide forecasting [42]. Similarly, MLPR has proven effective in predicting PM2.5 concentrations, outperforming the Long Short-Term Memory (LSTM) network [43], and has exhibited superior performance in simulating solar diffuse radiation [44]. Although LSTM models have been widely tested for sequential data predictions, the data used in this study involve high-dimensional features across nearly one hundred variables. LSTM encounters significant memory overhead when handling such datasets, especially with long time-series data, as it must store gradients and hidden states for every time step in memory. This results in increased training time and computational burden compared to MLPR.

Moreover, the sequential dependence in high-dimensional data can limit LSTM’s parallelization potential. LSTM models, with their larger parameter space, are also more prone to overfitting on high-dimensional datasets. If the temporal correlation between different atmospheric circulation features is weak, LSTM may not perform efficiently, leading to wasted computational resources. In scenarios where features are relatively independent across time steps, redundant computations at each step further increase the cost. By contrast, MLPR is well-suited for processing independent, non-sequential multi-dimensional features, as it captures feature relationships through fully connected layers. Given that the data in this study were optimized by Random Forest to retain relatively independent features, MLPR provides a more efficient solution by capitalizing on this structure, achieving better performance with lower computational costs.

Despite the improvements offered by individual models through parameter optimization, their fixed structures and limited parameter ranges restrict their adaptability to different basins. Coupled models offer a solution by combining models from various domains or scales, enabling a more comprehensive understanding of complex systems. These models account for interactions and feedback mechanisms across multiple factors, thereby enhancing analysis and prediction capabilities. For example, coupling the WetSpass-M and MODFLOW models resulted in higher accuracy in groundwater balance assessments compared to individual models [45]. In another study, a nested multi-coupling model was validated through experiments on artificial dams, demonstrating the feasibility and effectiveness of the approach [46]. Moreover, a wind-wave coupling model was optimized and validated using a hybrid wind-wave system, achieving high prediction accuracy and improving energy collection efficiency [47].

Given this, in this study, we combined the Random Forest (RF) algorithm, Support Vector Regression (SVR), and Multilayer Perceptron Regression (MLPR) to construct two medium- to long-term runoff coupled prediction models: RF-SVR based on machine learning and RF-MLPR based on deep learning. For a case-study basin, the RF module of the coupled model first selects features from high-dimensional teleconnection data based on the characteristics of runoff data sequences from different target hydrological stations to eliminate collinear and redundant variables from high-dimensional data and form feature subsets. Subsequently, different feature subsets selected for different hydrological stations are input into the subsequent modules (SVR or MLPR) of the coupled model. The overall coupled model is then optimized and iteratively trained through hyperparameter optimization methods. Finally, focusing on four typical hydrological stations in the Yalong River basin (YLRB), we investigate the influence of different prediction steps on runoff prediction accuracy and verify the effectiveness and applicability of the proposed coupled model in improving medium- to long-term runoff prediction accuracy. The main objectives of this study are: (1) to propose two high-accuracy coupled models (RF-SVR based on machine learning and RF-MLPR based on deep learning) that comprehensively consider multidimensional variables and can autonomously extract effective features; (2) to enrich the factor variables of medium- to long-term runoff prediction; (3) to identify the lag duration of atmospheric circulation affecting basin runoff; (4) to identify the optimal features for runoff prediction; (5) to validate the advantages of the coupled intelligent models over individual baseline models and the accuracy of the proposed coupled intelligent models at different prediction durations (1, 3, 6, and 12 months).

## 2 Materials and research methods

### 2.1 Study area and data source

#### 2.1.1 Study area

**Fig 1:**
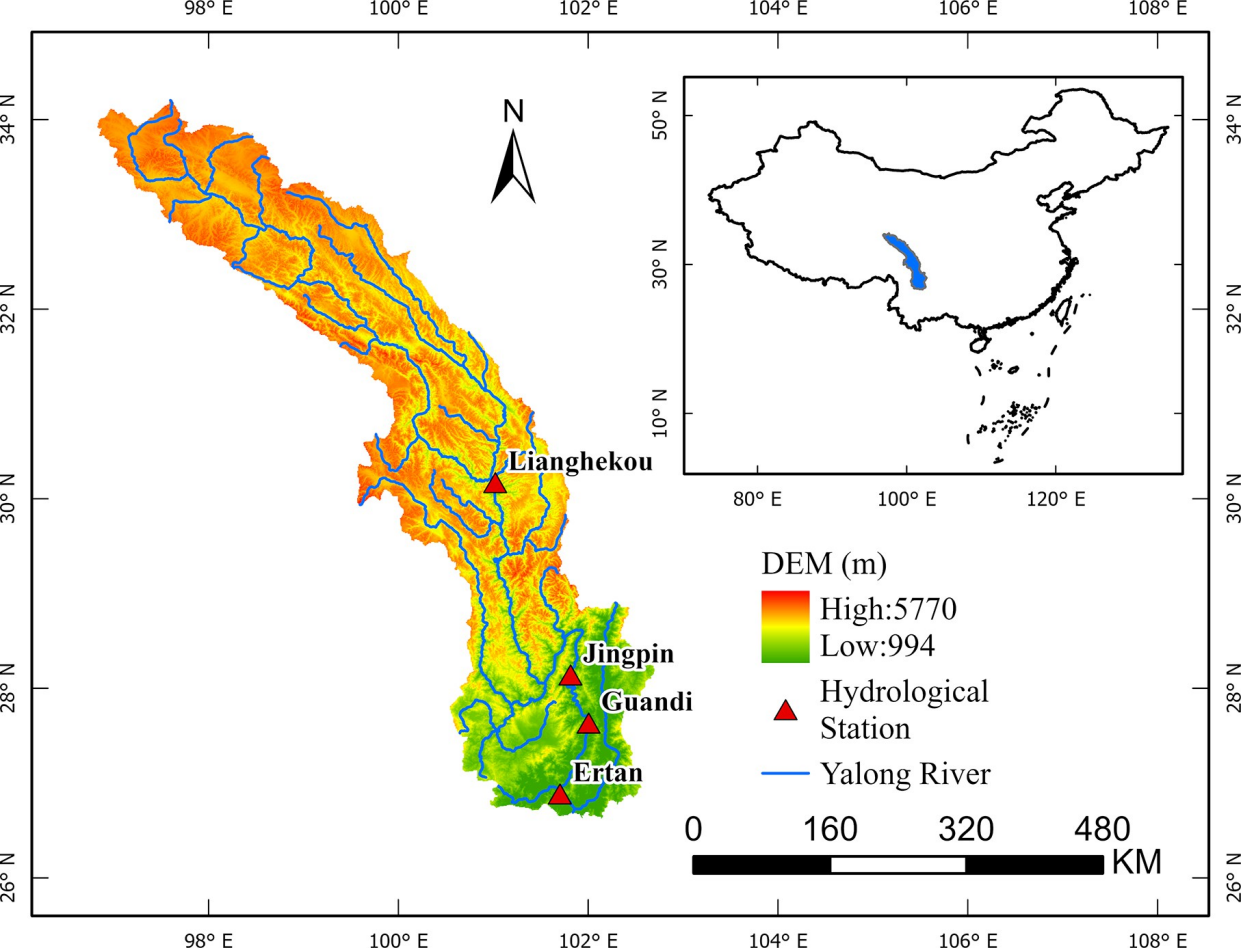
Location of the YLRB and distribution of 4 hydrological stations. (The data required to create this figure comes from the National Catalogue Service for Geographic Information: www.webmap.cn).

In order to develop a medium- to long-term runoff coupled prediction model that comprehensively considers and utilizes multi-source high-dimensional data variables, we chose the YLRB in southwestern China (see Fig 1) as a case study. The YLRB is located in the southwestern region of China and originates from the Kunlun Mountains within the Yushu Tibetan Autonomous Prefecture of Qinghai Province. The basin covers an area between 96°52′ to 102°48′ east longitude and 26°32′ to 33°58′ north latitude. It traverses parts of Sichuan Province, Yunnan Province, and the Tibet Autonomous Region before ultimately merging with the Jinsha River in Panzhihua City. The mainstem of the YaLong River is 1571 km long, with a total drop of 3830 m from its source to its mouth. The basin covers an area of 136,000 square kilometers and extends in a roughly north‒south direction. It forms a strip-like shape and represents the largest first-order tributary on the left bank of the Jinsha River.

As a significant hydropower base in China, the YLRB is endowed with abundant water resources. Its terrain is characterized by low elevations in the southeast and high elevations in the northwest. The variation in elevation is pronounced in the north‒south direction. Influenced by the high-altitude westerly atmospheric circulation and the southwestern monsoon, the climate changes markedly, resulting in diverse natural landscapes and ecosystems within the basin. The northern plateau of the basin experiences a dry and cold continental climate, with cold and dry temperatures and an average annual temperature of approximately 0°C. In contrast, the central and southern parts of the basin belong to a subtropical climate zone. In the same region, the climate is damp and rainy with lower temperatures at higher elevations, while the valley areas are characterized by clear and dry conditions with less rainfall and higher temperatures. The YLRB is characterized by numerous short tributaries, creating a feather-like distribution of its water system. Fig 1 illustrates the specific location of the YLRB.

Recent years have witnessed frequent occurrences of extreme high temperatures and heavy rainfall, leading to floods in the YLRB. These events have had a significant impact on the human living environment. Research on runoff prediction in the YLRB is of great significance for flood control and scheduling, scientific water resource planning, orderly development and utilization of water resources, and protection of the regional ecological environment.

#### 2.1.2 Data source

**Fig 2:**
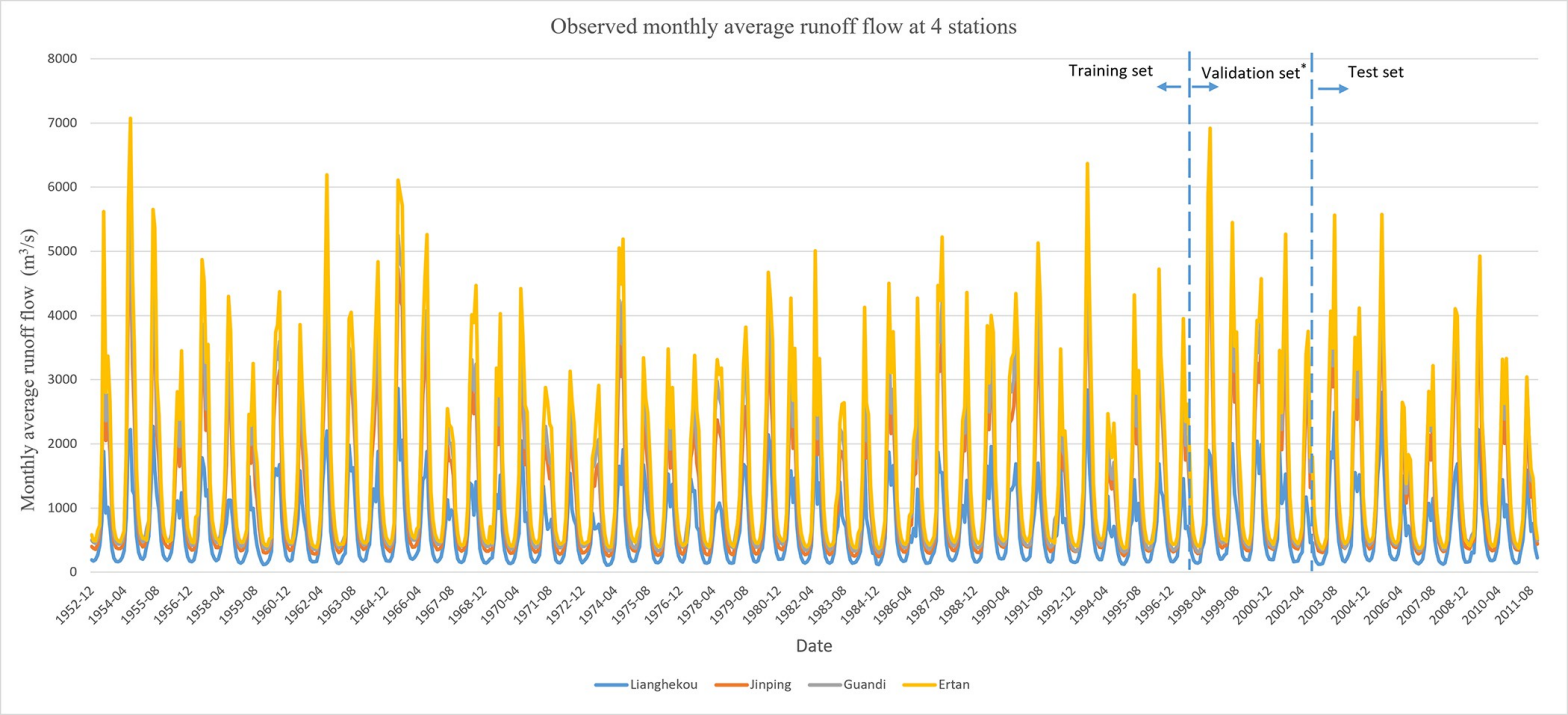
Monthly runoff observation values and variation in 4 hydrological stations in the YLRB. The asterisk * in the figure is used solely as an example for validation dataset verification. In the subsequent algorithm training process, the selection of the validation dataset is performed through random sampling, following the K-fold cross-validation method.

This study focuses on conducting research using monthly runoff data from four major hydrological stations within the YLRB. These stations include Lianghekou (LHK) Station, Jinping (JP) Station, Guandi (GD) Station, and Ertan (ET) Station. The distribution of these four stations can be observed in Fig 1. The runoff data comprise the monthly average runoff values recorded at these four hydrological stations from 1953 to 2011. The observed monthly runoff variations for the four stations are depicted in Fig 2, with different colors representing different stations.

The atmospheric circulation index data are sourced from the China National Climate Centre (http://cmdp.ncc-cma.net/cn/monitoring.htm), providing a total of 88 atmospheric circulation indices. For this study, 80 out of these 88 atmospheric circulation indices are selected as experimental data. The remaining 8 indices were excluded due to reasons such as missing values exceeding 80%, 0 values constituting more than 80%, or 999 values or -999 values constituting more than 80%. These columns were considered to lack practical significance for learning algorithms. Consequently, this study removed these abnormal and meaningless columns. Subsequent experimental calculations are based on the 80 atmospheric circulation indices after removing the ineffective columns. These indices are combined with the monthly average runoff data from the four hydrological stations in the YLRB for the research analysis.

### 2.2 Research methods

#### 2.2.1 Data preprocessing

Data preprocessing refers to the operations of cleaning, transforming, and integrating raw data before conducting data analysis and modelling, aiming to enhance data quality and applicability. Data preprocessing stands as a vital preliminary step in machine learning and deep learning and is essential for accurately and reliably extracting valuable information from data.

Due to the significant differences in the dimensional ranges among the various atmospheric circulation index values in this study, the normalization method is employed to standardize the dimensional representation of these indices.

The transformation method of numerical normalization is depicted as shown in Eq (1):

$$x_{new}=\frac{x-x_{min}}{x_{max}-x_{min}}$$ (1)

#### 2.2.2 K-fold cross validation and grid search cross validation

*2*.*2*.*2*.*1 K-fold cross validation*. K-fold cross-validation (K-CV) is a method for evaluating the generalization performance of machine learning models. It divides the original dataset into K equal and mutually exclusive subsets. In each iteration, one of these subsets is used as the testing dataset, while the remaining subsets are used as the training dataset. This process is repeated K times, each time using a different subset as the testing set. The performance metrics on the different testing sets are then averaged to provide the final performance indicator for the model. K-fold cross-validation effectively reduces the impact of randomness on model performance evaluation, better reflects the model’s performance on unseen data, avoids overfitting and underfitting, assists in selecting optimal features and model parameters, and enhances the stability and reliability of prediction results.

*2*.*2*.*2*.*2 Grid search cross validation*. Grid Search Cross Validation (GridSearchCV) is an algorithm used as a tool for hyperparameter tuning with the grid search method [48]. It can effectively train and learn from the dataset by combining K-fold Cross Validation (K-CV). GridSearchCV automates the search for all possible parameter combinations within the given parameter space and systematically evaluates and compares the performance of each combination, ultimately finding the best parameter combination. When using this function, by specifying the parameter space, evaluation function, and estimator and adjusting and optimizing based on practical considerations, the optimal performing model within the given parameter space can be obtained.

GridSearchCV, combined with K-CV, is used in this study for the hyperparameter optimization phase of model construction.

#### 2.2.3 Random forest algorithm

The incorporation of teleconnection variables as input factors in medium- to long-term runoff forecasting models has significantly enhanced both predictive accuracy and generalization capabilities [49, 50]. From a physical and causal standpoint, teleconnection variables provide a robust foundation for runoff prediction [51]. However, a critical challenge arises from the abundance of meteorological, hydrological, and large-scale climatic data, which often comes with a limited number of observational samples and a high-dimensional feature space. High-dimensional data typically contains substantial redundancy and irrelevant information, making it difficult to achieve satisfactory results when using raw data directly for classification or prediction tasks.

**Fig 3:**
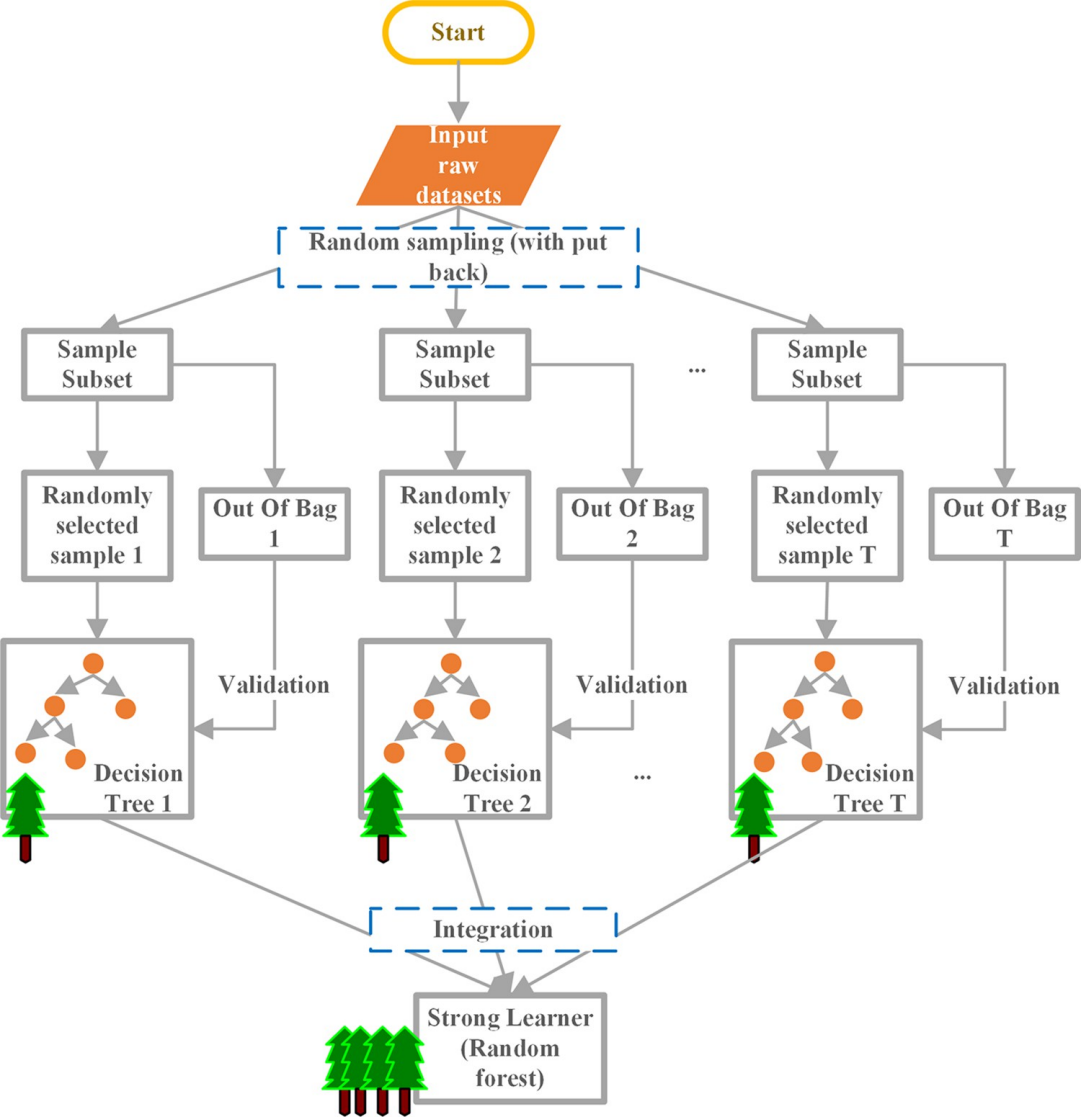
The basic process of feature selection of the random forest algorithm.

Given the prevalent redundancy and collinearity among high-dimensional atmospheric circulation teleconnection factors, this study applies the Random Forest algorithm to effectively select the most relevant feature variables that influence runoff at hydrological stations. The Random Forest algorithm assesses the contribution of each feature variable through the Variable Importance Measure (VIM), allowing for an accurate and efficient evaluation of each feature’s impact on the overall model performance [52]. As a robust and efficient method for feature selection in high-dimensional datasets, Random Forest integrates the Bagging technique from ensemble learning, which involves random sampling of both data and features. This approach mitigates overfitting and reduces correlations between individual decision trees, thus enhancing the algorithm’s ability to model nonlinear relationships and improve generalization. Originally proposed by Breiman [53], the detailed workflow of the Random Forest algorithm is illustrated in Fig 3.

The calculation of contribution magnitude can be evaluated using metrics such as the Gini index or out-of-bag data error. During the construction of decision trees, the Gini index is employed to select the optimal features and split points, aiming to minimize the Gini index of child nodes. By iteratively selecting the features and split points that minimize the Gini index for node splitting, a decision tree model with high purity is constructed. The formula for the Gini index is as follows:

$$Gini(p)=1-\sum_{i=1}^{k}p_{k}^{2}$$ (2)

In Eq (2), k represents the number of features. P_k_ signifies the proportion of feature k within the node.

#### 2.2.4 Support Vector Regression

Support Vector Regression (SVR) demonstrates significant advantages in handling high-dimensional and nonlinear data [54]. It achieves nonlinear mapping through the use of kernel techniques, while leveraging the sparsity of support vectors to enhance computational efficiency. SVR also exhibits excellent generalization ability and robustness, making it suitable for datasets of various scales. Additionally, it avoids the need to compute inverse matrices, which makes it an ideal choice for solving such problems.

**Fig 4:**
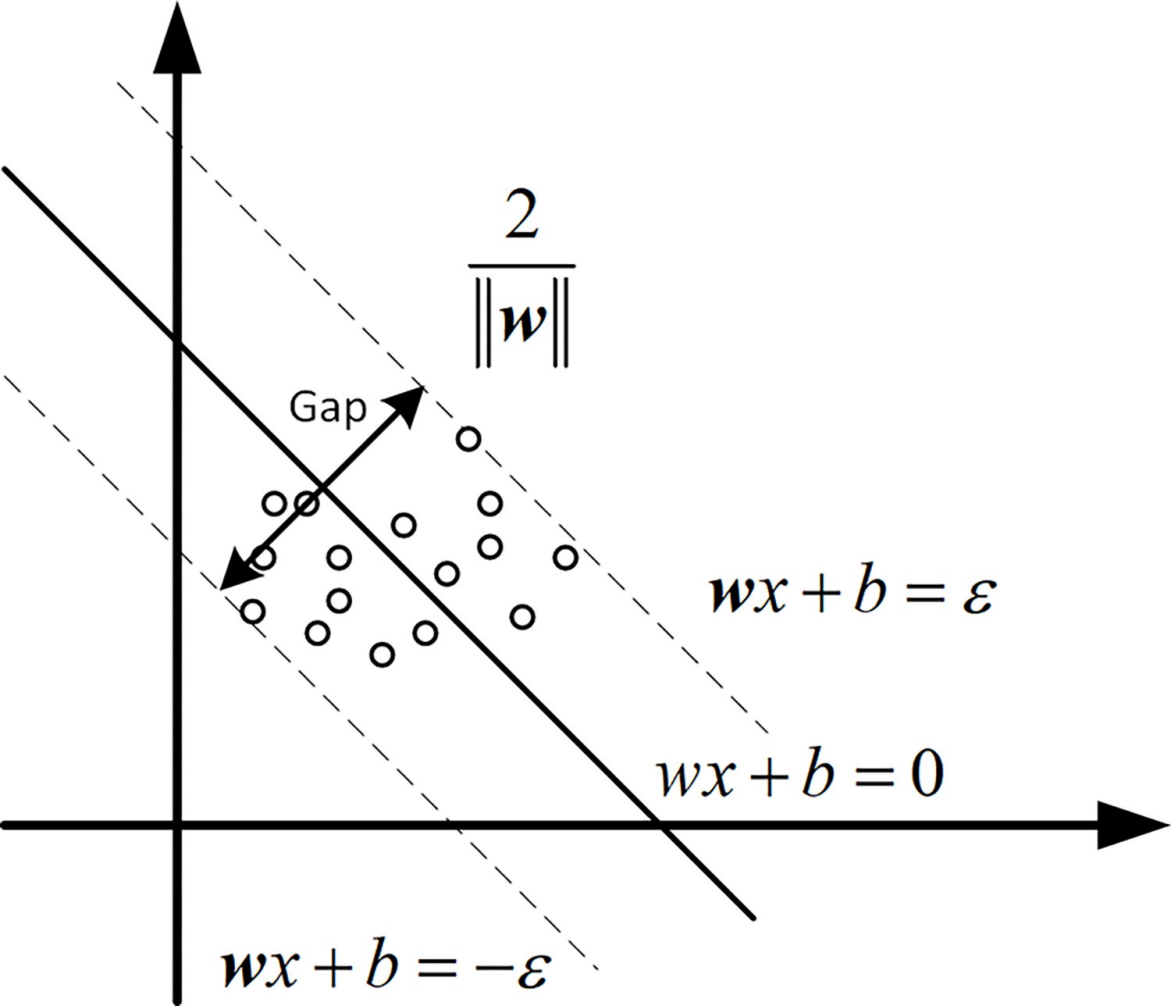
Schematic diagram of the basic principles of SVR.

**Fig 5:**
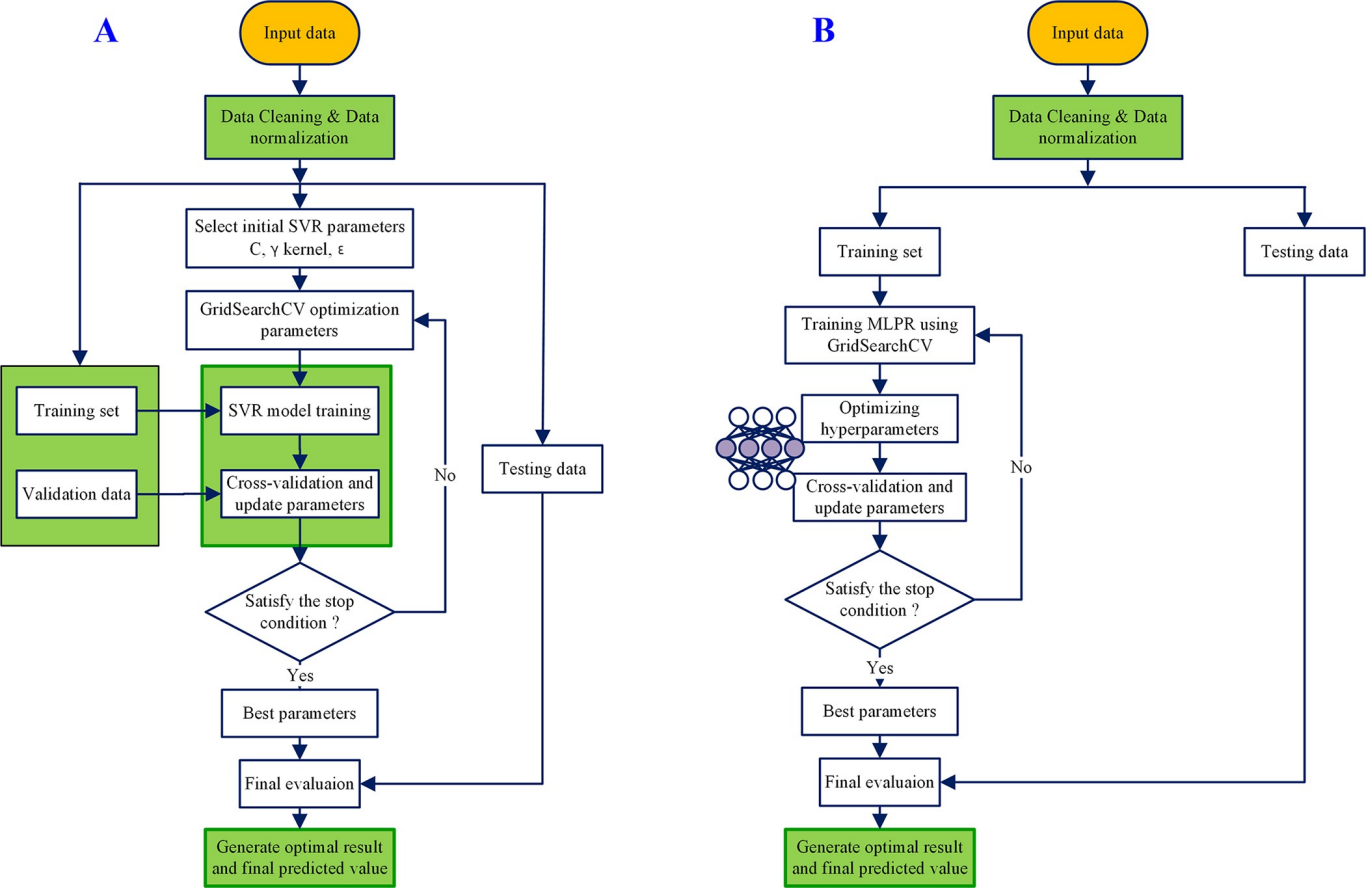
SVR and MLPR algorithm flow optimized by GridSearchCV.

SVR is widely used to solve regression problems and is particularly suitable for handling nonlinear and high-dimensional data. By introducing kernel functions, penalty parameters (also known as regularization parameters), gamma parameters, and tolerance deviation epsilon, SVR maps the feature variables into a high-dimensional space. It constructs a family of hyperplane functions F(X) in such a way that the sum of distances from all sample points to a specific function hyperplane in F(X) is minimized. This specific hyperplane is the sought optimal hyperplane, as illustrated in Fig 4 Parameter optimization is a key issue in the modelling training process of SVR. In this study, GridSearchCV is utilized for parameter tuning in the SVR modelling process, and its operational process is illustrated in Fig 5A.

#### 2.2.5 Multilayer perceptron regressor neural network

The Multilayer Perceptron Regression (MLPR) model excels in handling large-scale high-dimensional data, as it can automatically extract key features and reveal potential nonlinear relationships between independent variables and the target variable. This model is particularly well-suited for capturing complex nonlinear mappings between input features and continuous target variables, thereby demonstrating its unique advantages in solving regression problems.

MLPR is a regression model based on a multilayer neural network, which is a significant type of artificial neural network (ANN) model [55–57]. The fundamental structure of MLPR consists of multiple neurons organized in layers, forming a multilayer neural network. Each neuron has an input and an output, and the connection weights between them can be auto-adjusted through training (as shown in Fig 5B). MLPR consists of multiple layers of neurons, with each layer being fully connected to its adjacent layers. Nonlinearity is introduced through activation functions such as sigmoid, tanh, and ReLU. The MLPR algorithm uses forward propagation combined with activation functions to generate predicted values as outputs. It utilizes backpropagation along with gradient descent algorithms to update the model’s weights and biases, thereby enhancing its fitting capacity and generalization performance. The selection and optimization of parameters required by MLPR are crucial for its performance. For the hyperparameters needed by MLPR (such as the number of hidden layers, neurons per layer, learning rate, activation function, loss function, batch size, iteration count, and whether dropout is needed), this study employs the hyperparameter tuning method GridSearchCV for handling.

#### 2.2.6 Pearson correlation coefficient method

The Pearson correlation coefficient (PCC), also known as the Pearson product-moment correlation coefficient (PPMCC), is widely utilized in statistical and data analysis. It serves as a statistical metric to measure the strength of relationships between any two variables, X and Y. This coefficient aids in discovering and explaining patterns and trends within data, playing a crucial role in comprehending the correlations between variables and in conducting prediction and modeling tasks. In this research work, we employ this method to explore and analyze the correlation between various atmospheric circulation indices and monthly runoff at hydrological stations.

### 2.3 Model evaluation metrics

The selection of model evaluation metrics not only impacts the fitting and predictive performance of the models but also determines their generalization ability on unseen data. To comprehensively assess the fitting and generalization capabilities of the models, this study employs six metrics for model evaluation: coefficient of determination (R-Square or R^2^), Nash-Sutcliffe efficiency coefficient (NSE), root mean square error (RMSE), mean absolute error (MAE), mean absolute percentage error (MAPE), and mean squared logarithmic error (MSLE). Below are the computational formulations for the six error metrics.

$$R^{2}(y,\hat{y})=1-\frac{\sum_{i=1}^{N}{\left(y_{i}-{\hat{y}}_{i}\right)}^{2}}{\sum_{i=1}^{N}{\left(y_{i}-\bar{y}\right)}^{2}}$$ (3)

$$NSE=\frac{{\left[\sum_{i=1}^{n}\left(y_{i}-\bar{y}\right)({\hat{y}}_{i}-\bar{\hat{y}})\right]}^{2}}{\sum_{i=1}^{n}{\left(y_{i}-\bar{y}\right)}^{2}\sum_{i=1}^{n}{\left({\hat{y}}_{i}-\bar{\hat{y}}\right)}^{2}}$$ (4)

$$RMSE(y,\hat{y})=\sqrt{\frac{\sum_{i=1}^{n}{\left(y_{i}-{\hat{y}}_{i}\right)}^{2}}{n}}$$ (5)

$$MAE(y,\hat{y})=\frac{\sum_{i=1}^{n}|y_{i}-{\hat{y}}_{i}|}{n}$$ (6)

$$MAPE(y,\hat{y})=\frac{100\%}{n}\sum_{i=1}^{n}|\frac{y_{i}-{\hat{y}}_{i}}{y_{i}}|$$ (7)

$$MSLE(y,\hat{y})=\frac{1}{n}\sum_{i=1}^{n}{\left(\log(1+y_{i})-\log(1+{\hat{y}}_{i})\right)}^{2}$$ (8)

where *y_i_* represents the *i*-th actual observed value. ${\hat{y}}_{i}$ represents the corresponding model-predicted value. $\bar{y}$ denotes the mean of the actual observed values. n represents the number of samples used by the model.

### 2.4 Coupling model running pseudocode

Table 1 presents the pseudocode of the coupled models (RF-SVR and RF-MLPR). The processing flow of the input dataset by the coupled models is as follows:

First, hyperparameters for each model are initialized. Then, the system automatically preprocesses the atmospheric circulation indices and other data, dividing the dataset into training, validation, and test sets, which are fed into the respective models. For each hydrological station, feature selection is performed to gradually eliminate redundant features and collinear variables. The selected features are then input into the coupled models, and iterative training is conducted using GridSearchCV and K-fold cross-validation. This process continues until the models meet the predefined convergence criteria. Finally, the trained models are used to predict the target data, followed by performance evaluation and analysis of the prediction results.

**Table 1:** Pseudo code for coupling model execution process.

**Pseudocode for RF-MLPR and RF-SVR Coupled Model**
**Input:** Atmospheric circulation index dataset and monthly runoff observation data for training, along with atmospheric circulation data corresponding to the target monthly runoff to be predicted.
**Output:** Predicted runoff for future months based on the model.
**Start:**
Initialize RF, SVR, and MLPR models with hyperparameters.
Load and preprocess the dataset (YLRB runoff data and atmospheric indices).
for station in hydrological_stations:
#Step 1: Feature Selection using Random Forest
selected_features = RF.fit_transform(data[station])
#Step 2: Model Training and Validation
Train RF-SVR and RF-MLPR using K-fold cross-validation:
for model in [RF-SVR, RF-MLPR]:
GridSearchCV(model, param_grid).fit(train_data)
Evaluate whether the performance of the trained model meets the preset criteria.
If not, proceed with the next iteration.
#Step 3: Model Prediction
predictions = model.predict(test_data)
Evaluate model performance (R^2^, NSE, RMSE, etc.)
**End.**

### 2.5 Research framework

**Fig 6:**
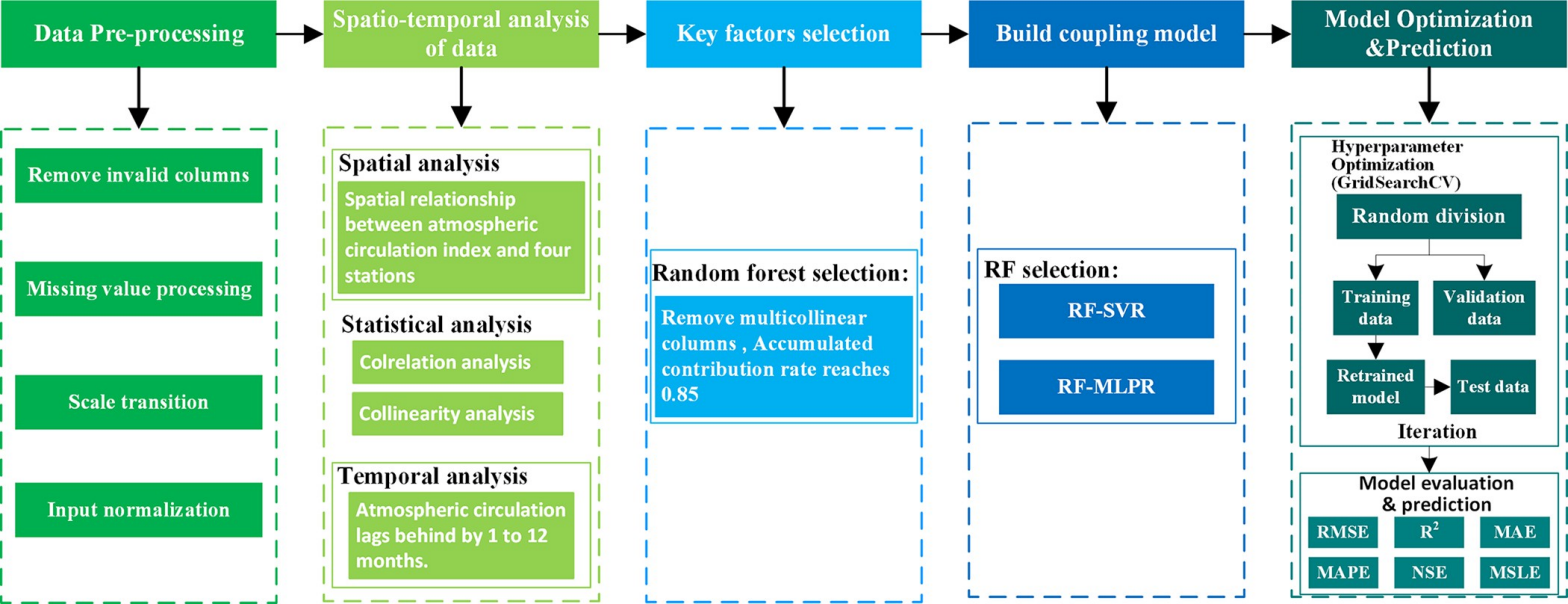
Flowchart of the integrated framework developed for runoff prediction.

Given the lag effect of atmospheric circulation indices on runoff, this study constructs a machine learning-based RF-SVR coupled intelligence model and a deep learning-based RF-MLPR coupled intelligence model, taking into account the characteristics of monthly runoff data in the YLRB, the features of atmospheric circulation indices, and the basic principles of the aforementioned algorithms. The overall framework and steps are illustrated in Fig 6.

1. **Multi-source data fusion and preprocessing:** Firstly, integrate the runoff observation data from four hydrological stations in the YLRB and 88 atmospheric circulation index data (1952–2011). Then, perform preprocessing steps on the integrated data, which include but are not limited to: removing invalid columns, dealing with missing values, scaling transformation, and input normalization, etc.
2. **Investigating temporal and spatial relationships:** Explore the temporal and spatial relationship between the observed runoff data and the preprocessed atmospheric circulation index data to determine the lag period of the impact of atmospheric circulation indices on the runoff volume at the basin’s hydrological stations.
3. **Data alignment and feature selection:** Align the atmospheric circulation indices with the runoff data from the four hydrological stations along the time dimension, based on the lag period determined in the previous step. The random forest algorithm was used for feature selection among the 88 atmospheric circulation indices to identify those that significantly impact runoff. Redundant and collinear variables were removed, considering issues of collinearity, nonstationarity, and nonlinear relationships. Appropriate feature selection criteria were established to obtain the final optimized variable datasets for the four hydrological stations.
4. **Model construction:** Construct the machine learning-based RF-SVR coupled model and the deep learning-based RF-MLPR coupled model. Initial parameters for both models were set.
5. **Model optimization and evaluation:** Divide each of the four datasets randomly into training and validation sets. Continuously optimize and iterate the models built in step 4 using the GridSearchCV hyperparameter tuning tool until the pre-set stopping conditions are reached. Eight final trained models (RF-SVR and RF-MLPR for each station) were obtained. Ultimately, use the optimized models to predict the test datasets for each station, and employ the six evaluation metrics shown on the right side of Fig 6 to assess the models’ performance.

This model framework is applied to predict runoff at the four hydrological stations in the YLRB, verifying its effectiveness and potentially serving as a reference for medium- to long-term runoff prediction in other basins. The research framework is shown in Fig 6.

## 3 Results

### 3.1 Analysis of spatial-temporal correlations between atmospheric circulation indices and runoff

#### 3.1.1 Spatial analysis of the relationship between atmospheric circulation indices and runoff

This section aims to conduct an in-depth spatial analysis of the correlation between runoff at the four hydrological stations in the YLRB and atmospheric circulation indices. By studying the correlation coefficients between each station and 80 atmospheric circulation indices, we can further identify the representative hydrological stations in the YLRB that are most correlated with the atmospheric circulation indices. This forms the basis for determining the lag duration of the influence of atmospheric circulation on runoff in the YLRB.

**Fig 7:**
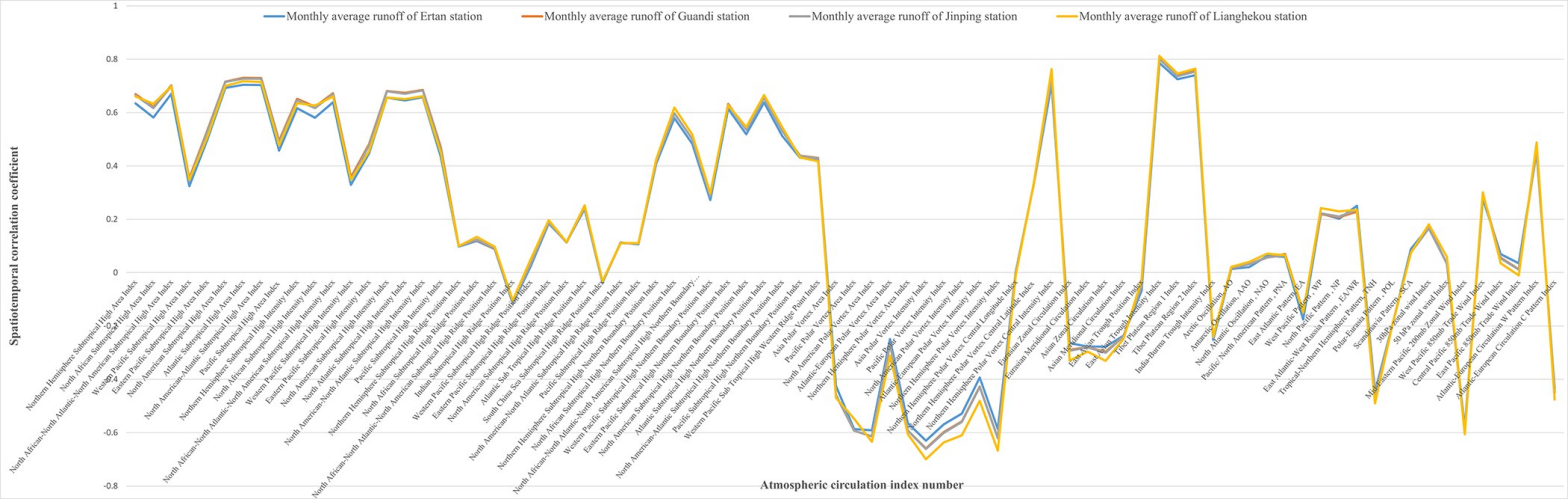
The spatial distribution map of the correlation between atmospheric circulation indices and runoff at four hydrological stations in the YLRB.

We selected four key hydrological stations in the YLRB for our study: LHK Station, JP Station, GD Station, and ET Station. The monthly average runoff data from these four hydrological stations were subjected to Pearson correlation analysis with 80 atmospheric circulation indices. Fig 7 illustrates the correlation calculation results between various hydrological stations and multiple atmospheric circulation indices, where the horizontal axis represents different atmospheric circulation indices. Fig 7 illustrates the correlation calculation results between various hydrological stations and multiple atmospheric circulation indices, where the horizontal axis represents different atmospheric circulation indices.

From Fig 7, it can be observed that there is a distinct spatial distribution pattern in the correlation between the YLRB runoff and atmospheric circulation indices. Specifically, there is a stronger correlation between upstream section runoff and atmospheric circulation indices, while the correlation weakens downstream, exhibiting a gradual decrease in overall correlation from upstream to downstream. Specifically, the hydrological station LHK upstream of the YLRB shows the highest correlation between monthly average runoff and various atmospheric circulation indices. Subsequently, the downstream stations, JP, GD, and ET, exhibit decreasing correlations with the atmospheric circulation indices in the same order. Among these four stations, ET Station, situated furthest downstream, has the weakest correlation between monthly average runoff and atmospheric circulation indices.

Taking into account the geographical distribution of the stations in the YLRB, the reasons for these observations can be analysed as follows:

1. The upstream region of the basin is situated in the plateau area of the Kunlun Mountains. The process of river runoff evolution is relatively natural, with simple and stable runoff generation and propagation relationships.
2. The distribution of water systems in the upstream area is relatively concentrated, with fewer branches and intersections, resulting in a simpler topology of the water system. This simplicity contributes to a relatively stable and straightforward evolution pattern of river runoff.
3. The upstream region of the basin experiences less human interference and lacks large-scale cities and human construction projects. Hydrological processes in the basin are primarily influenced by natural factors such as topography, soil, precipitation, and evaporation. This natural influence leads to a simpler and more stable runoff evolution pattern in the upstream area.

#### 3.1.2 Temporal variation analysis of the correlation between atmospheric circulation indices and runoff

In this section, we will analyse the spatial-temporal correlation between runoff observation data from the four hydrological stations in the YLRB and the preprocessed atmospheric circulation index data. By studying the correlation coefficients at different time lags, we can determine the lag duration at which atmospheric circulation indices impact runoff, providing crucial information for subsequent model construction and analysis.

As shown in Section 3.1.1, among the four hydrological stations in the YLRB, there exists a significant correlation between atmospheric circulation indices and the monthly average runoff at the LHK hydrological station. Given this, we consider studying the monthly average runoff at LHK station as the research subject to explore the temporal relationship between YLRB runoff and atmospheric circulation indices, which is highly feasible.

**Fig 8:**
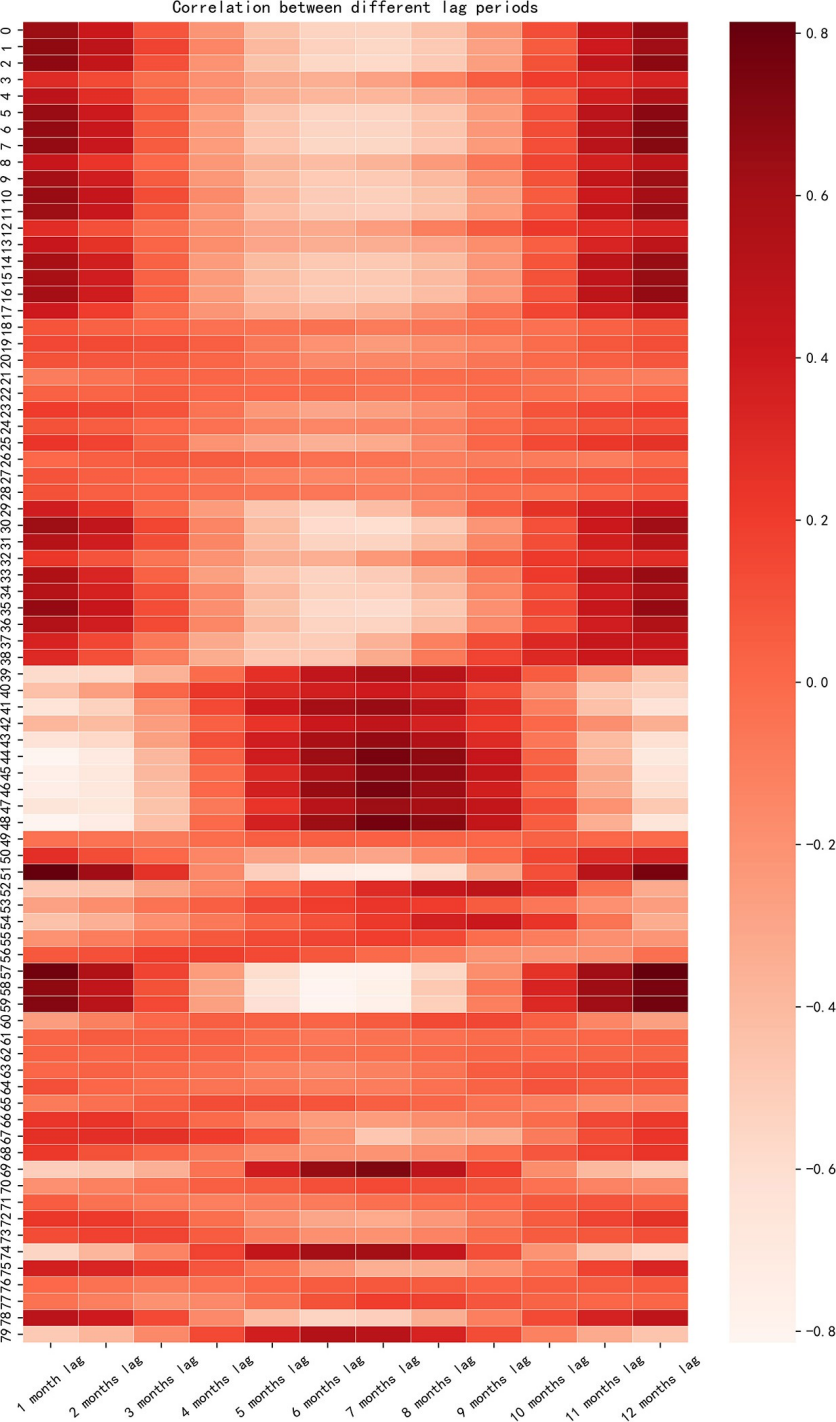
Correlation heatmap of atmospheric circulation indices and monthly average runoff at LHK hydrological station with different lag periods.

For monthly runoff prediction, a complete hydrological cycle consists of 12 months. Considering that the impact of atmospheric circulation indices on runoff might exhibit lag effects, for comprehensive analysis, we selected LHK station as the analysis object and conducted Pearson correlation analysis between the current month’s runoff and the preceding 1 to 12 months’ atmospheric circulation indices. After calculating the correlation coefficients, we generated a correlation heatmap (as shown in Fig 8). In the heatmap, darker colors represent higher positive correlations, lighter colors represent stronger negative correlations, and the colors tend to transition between darker and lighter shades for smaller absolute correlation values. The numbering on the left side of Fig 8 corresponds sequentially to the atmospheric circulation indices shown on the horizontal axis of Fig 7.

From Fig 8, it is evident that there is a strong correlation between the monthly average runoff at LHK station and the atmospheric circulation index of the previous month, while the correlation with atmospheric circulation indices of other months is relatively low. Specifically, the maximum correlation is achieved with a lag period of one month. As the lag period increases, the correlation gradually decreases until reaching the lowest point at lag periods of six and seven months. Beyond this, with a continuous increase in the lag period up to twelve months, the correlation starts to rise again. Analysing this in relation to time and meteorology, the reasons are as follows:

1. The runoff process exhibits a cyclic nature with a period of one year, corresponding to Earth’s orbit around the Sun.
2. When the lag period is twelve months, the atmospheric circulation index corresponds to the runoff of the previous January, which is one year before. Due to the cyclic nature of runoff over twelve months, the twelfth month’s atmospheric circulation index lag period corresponds to the runoff of the previous cycle’s January. This again highlights the most significant impact and highest correlation at a lag period of one month.

The results and analysis above suggest that for medium- to long-term runoff prediction in the YLRB, selecting a lag period of one month for atmospheric circulation indices is an appropriate choice. Atmospheric circulation from even earlier months has a relatively minor impact on the current month’s runoff.

### 3.2 Screening atmospheric circulation indices using random forest method

**Fig 9:**
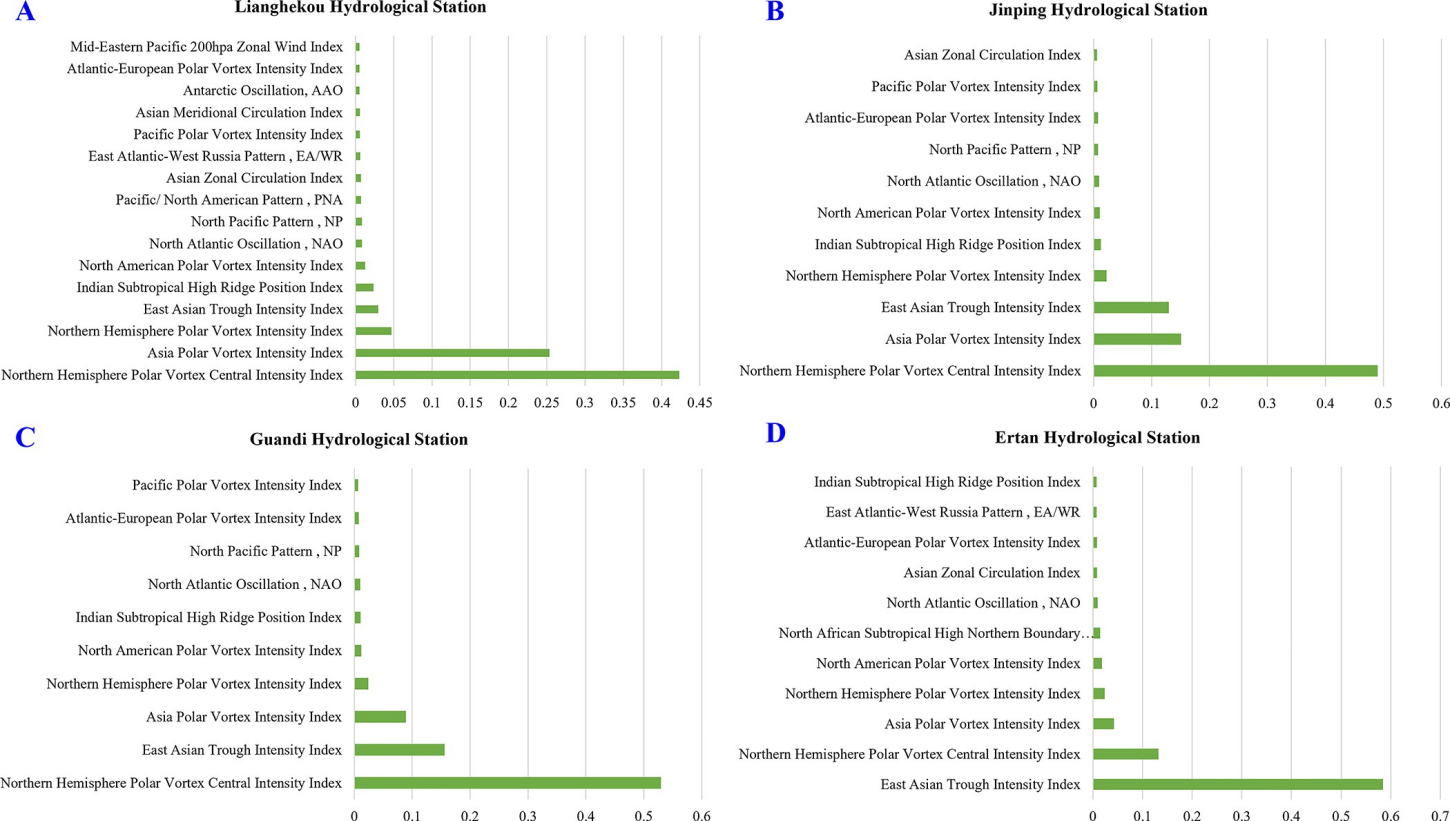
Results of RF algorithm screening factors.

Based on the experimental analysis results from the previous section, we lagged the atmospheric circulation index data by one month and temporally aligned it with the runoff data from the four hydrological stations in the YLRB. Subsequently, we inputted these aligned data into the random forest algorithm for computation, and the feature selection results obtained are presented in Fig 9.

We utilized an 85% cumulative contribution rate as the selection criterion and employed the random forest algorithm to perform feature selection among 80 atmospheric circulation indices. For the four hydrological stations in the YLRB, we identified sets of influencing factors. This process resulted in four sets of selected feature variables. Subsequently, for each selected set of variables at each station, we constructed bar charts similar to Fig 9, with feature importance contribution rate values on the horizontal axis and feature names on the vertical axis, facilitating further analysis.

Observations from Fig 9 lead to the following conclusions:

1. With a cumulative contribution rate of 85%, LHK station covers 16 atmospheric circulation indices, JP station covers 11, GD station covers 10, and ET station covers 11. There is significant overlap in the indices covered by each station, demonstrating consistency in spatial distribution among these four stations.
2. Analysing the preferred atmospheric circulation indices for each station reveals notable numerical differences between various circulation indices. Differences in the contribution rates of circulation indices within some stations are up to threefold. This reflects the effectiveness of random forests in handling multicollinearity among independent variables during feature selection and its accuracy in identifying variables that significantly affect the target variable.
3. By observing the number of selected variables for these four stations, it can be seen that the random forest algorithm selects a larger number of circulation indices for LHK station, while the number of selected circulation indices decreases gradually for downstream stations (except for ET station). This demonstrates that the random forest algorithm efficiently filters out critical feature variables that have actual impacts on each station. Specifically, LHK, being the first hydrological station upstream, is influenced by a broader range of atmospheric circulation indices. As stations move downstream and human factors become more prominent, the influence of human activities reduces and decreases dependence on atmospheric circulation indices.

Furthermore, from Fig 9, it is evident that the Northern Hemisphere Polar Vortex Central Intensity Index, Asia Polar Vortex Intensity Index, Northern Hemisphere Polar Vortex Intensity Index, and East Asian Trough Intensity Index are the four most influential atmospheric circulation indices in the YLRB. These four indices also rank among the top four indices for each hydrological station. Considering the geographical location of the YLRB between 96°52′E to 102°48′E longitude and 26°32′N to 33°58′N latitude and analysing these four circulation indices further reflects the effectiveness of the random forest approach in selecting key atmospheric circulation indices.

### 3.3 Model hyperparameter configuration and selection

For machine learning or deep learning models, obtaining accurate prediction results requires considering the influence of model hyperparameters. Hyperparameters are crucial in determining the convergence speed, performance, and generalization ability of the algorithm model. To select appropriate and excellent hyperparameters, this section provides multiple suitable and excellent candidate parameters for each parameter of the coupled machine learning model RF-SVR and the coupled deep learning model RF-MLPR. Based on this, for the hyperparameter tuning of the RF-SVR and RF-MLPR models, we employ the GridSearchCV method introduced in Section 2.2.2. The operating principle of GridSearchCV involves iterating through all possible combinations of hyperparameters, evaluating the performance of each combination through cross-validation, and ultimately selecting the best-performing parameter combination as the optimal hyperparameter setting for the model. In both training phases of the two models in this study, 85% of the original runoff data sequence is used as a shared training and validation set (with 10-fold cross-validation), while the remaining 15% serves as an independent test set.

Table 2 presents the hyperparameter settings used in the training and prediction processes of the RF-SVR model, as well as the final determined best parameter values for the model. It can be observed that for the choice of the feature mapping kernel function in the RF-SVR model, GridSearchCV selected the widely adaptable radial basis function (RBF), which is capable of better handling nonlinear relationships and data distributions. When selecting the Gamma function, the Scale function was employed to ensure data-scale consistency and simplify the hyperparameter selection process, thereby accelerating the model’s convergence speed. Looking at the variations in the numerical values of the regularization parameter C and the tolerance bias Epsilon across the four hydrological stations, it can be inferred that the tolerance biases among the four stations are dimensionally consistent, suggesting the presence of some common characteristics among these stations. Additionally, the required number of iterations for each station to achieve the final model results varies, indicating that each station has its own unique characteristics.

**Table 2:** Major hyperparameters of RF-SVR.

RF-SVR Hyperparameter	Parameter value			
	LHK	JP	GD	ET
**C**	1000	1500	3000	2250
**Epsilon**	100	175	100	201
**Gamma**	Scale	Scale	Scale	Scale
**Kernel**	RBF	RBF	RBF	RBF
**Iteration count**	600	800	500	700
**Convergence tolerance**	1*10^−7^	1*10^−7^	1*10^−7^	1*10^−7^

Similar to **Tables 2 and 3,** also presents the hyperparameter settings used in the fitting and prediction processes of the RF-MLPR model, along with the final determined best parameter values for the model (since the RF-SVR and RF-MLPR models have different natures, their parameter items are diverse). From the table, several observations can be made:

1. **Activation Function Choice:** The model opted for the rectified linear unit (ReLU) function as the activation function. ReLU is computationally efficient, possesses strong nonlinear expressive power, and effectively mitigates the vanishing gradient problem.
2. **Number of Hidden Layers:** Depending on the characteristics of the runoff data and independent variable data for each of the four hydrological stations–LHK, JP, GD, and ET–the model selected 7 layers, 6 layers, 7 layers, and 6 layers, respectively, for the neural network’s hidden layer count.
3. **Optimization Algorithm:** The model chose the limited-memory Broyden-Fletcher-Goldfarb-Shanno (L-BFGS) algorithm for neural network optimization. L-BFGS is memory-efficient, converges rapidly and is suitable for handling large-scale data. It excels in efficiently solving nonlinear optimization problems, making it particularly suitable for optimizing and improving target variables affected by multiple factors.
4. **Iteration Count and Tolerance:** The default iteration count of 15,000 was employed, and the tolerance was set to 1e-7.

**Table 3:** Major hyperparameters of RF-MLPR.

RF-MLPR Hyperparameter	Parameter value			
	LHK	JP	GD	ET
**Activation function**	ReLU	ReLU	ReLU	ReLU
**Learning rate**	0.0001	0.0001	0.0003	0.0005
**Hidden layer**	7	6	7	6
**Optimization algorithm**	L-BFGS	L-BFGS	L-BFGS	L-BFGS
**Batchsize**	128	128	128	128
**Iteration count (Default)**	15000	15000	15000	15000
**Convergence tolerance**	1*10^−7^	1*10^−7^	1*10^−7^	1*10^−7^

These parameter selections highlight the model’s efforts to optimize its architecture and learning process based on the specific characteristics and requirements of the problem at each station.

### 3.4 Comparison and analysis of prediction results between coupled intelligent models and baseline models

To investigate the impact of coupled models compared to single models on medium- to long-term runoff prediction, this section designed comparative experiments between the coupled models (RF-SVR and RF-MLPR) and single models (SVR and MLPR). The prediction results of the test set are shown in Tables 3 and 4. We selected six metrics, including R-Square, NSE, RMSE, MAE, MAPE, and MSLE, for quantitative evaluation and comparative analysis of the overall coupled models and baseline models. To ensure a fair comparison of the practical performance of each module, the comparative experiments established the following models: SVR, RF-SVR, MLPR, and RF-MLPR. These corresponding models in the comparative experiments were conducted under the same hyperparameter settings (see Section 3.3), data set partition, and experimental conditions. The introduction of all evaluation metrics has been detailed in Section 2.3. The experimental results, as shown in Tables 4 and 5, present the values of these metrics obtained through 10-fold cross-validation using the respective models and averaged for analysis.

**Table 4:** The ablation study presents the comparison of the coupled model and the baseline model in terms of the same evaluation metrics at the LHK and JP hydrological stations.

Evaluation Indicators	LHK				JP			
	SVR	RF-SVR	MLPR	RF-MLPR	SVR	RF-SVR	MLPR	RF-MLPR
**R-Square**	0.6792	**0.7550±0.0005**	0.7036	**0.7834±0.0005**	0.6369	**0.7235±0.0005**	0.6708	**0.7710±0.0005**
**NSE**	0.6846	**0.7610±0.0006**	0.7087	**0.7892±0.0006**	0.6423	**0.7307±0.0006**	0.6803	**0.7789±0.0006**
**RMSE**	341.8323	**260.9348±2**	303.6378	**245.3119±2**	601.3359	**469.5169±2**	590.3090	**424.6687±2**
**MAE**	238.3268	**169.1202±0.5**	218.7850	**160.8181±0.5**	383.5630	**315.4585±0.5**	369.3606	**273.9098±0.5**
**MAPE**	0.3479	**0.2932±0.0002**	0.2708	**0.2357±0.0002**	0.3153	**0.2384±0.0002**	0.2689	**0.1233±0.0002**
**MSLE**	0.1433	**0.1103±0.0001**	0.1208	**0.0756±0.0001**	0.1278	**0.0791±0.0001**	0.0894	**0.0513±0.0001**

**Note:** The units of measurement for RMSE and MAE in the table are m^3^/s. Both RF-SVR and RF-MLPR were performed in 200 replicate trials due to the random initial weights of network, thus the metrics were expressed as the mean ± standard deviation.

**Table 5:** The ablation study presents the comparison of the coupled model and the baseline model in terms of the same evaluation metrics at the GD and ET hydrological stations.

Evaluation Indicators	GD				ET			
	SVR	RF-SVR	MLPR	RF-MLPR	SVR	RF-SVR	MLPR	RF-MLPR
**R-Square**	0.6787	**0.8020±0.0005**	0.7153	**0.8035±0.0005**	0.6368	**0.7644±0.0005**	0.6592	**0.7650±0.0005**
**NSE**	0.6825	**0.8066±0.0006**	0.7213	**0.8101±0.0006**	0.6389	**0.7702±0.0006**	0.6643	**0.7721±0.0006**
**RMSE**	636.3489	**514.0621±2**	620.3271	**447.5975±2**	798.4798	**648.2849±2**	779.5903	**420.7987±2**
**MAE**	389.8963	**308.7004±0.5**	381.9365	**303.8098±0.5**	485.7863	**405.6246±0.5**	456.8376	**249.9427±0.5**
**MAPE**	0.2697	**0.1903±0.0002**	0.2509	**0.1833±0.0002**	0.2615	**0.2268±0.0002**	0.2383	**0.1985±0.0002**
**MSLE**	0.0798	**0.0582±0.0001**	0.0722	**0.0578±0.0001**	0.0943	**0.0761±0.0001**	0.0873	**0.0638±0.0001**

**Note:** The units of measurement for RMSE and MAE in the table are m^3^/s. Both RF-SVR and RF-MLPR were performed in 200 replicate trials due to the random initial weights of network, thus the metrics were expressed as the mean ± standard deviation.

R-Square is an effective indicator for measuring model fitting goodness and generalization performance. A value closer to 1 indicates better predictive performance of the model, and it is widely used in prediction models in fields such as hydrology, climate, and environment. Tables 4 and 5 list the Prediction Results of the comparative experiments for various models at four hydrological stations. We can observe that at the LHK hydrological station, the coupled model RF-SVR improves the R-Square fitting goodness indicator by approximately 11.15% compared to the baseline model SVR. The R-Square indicators at the JP, GD, and ET hydrological stations show improvements of 13.5%, 18.1%, and 20%, respectively. Similarly, at the LHK hydrological station, the coupled model RF-MLPR improves the R-Square indicator by 11.3% compared to the baseline model MLPR. At the JP, GD, and ET hydrological stations, RF-MLPR shows improvements over MLPR of approximately 14.9%, 12.3%, and 16.04%, respectively. The enhancement of the R-Square indicator values in the coupled models relative to the baseline models indicates that the constructed coupled models in this study fully leverage the advantages of each module in the coupled model, effectively extracting significant feature variables that have a significant impact on the target hydrological stations, thereby improving prediction accuracy.

NSE is an important indicator used to assess the degree of fit between model predictions and observed values, and it is widely applied in hydrology, environmental, and climate prediction fields. A higher NSE value, closer to 1, indicates better predictive performance of the model. In Tables 3 and 4, we list the NSE values obtained from the testing datasets at four typical hydrological stations. Upon observation, it is evident that during the testing period, RF-MLPR achieved the highest R^2^ values (0.7834, 0.7710, 0.8035, and 0.7650) and NSE values (0.7892, 0.7789, 0.8101, and 0.7721) across all four stations. Following closely are the RF-SVR models, with R^2^ values of 0.7550, 0.7235, 0.8020, and 0.7644, and NSE values of 0.7610, 0.7307, 0.8066, and 0.7702, respectively. However, the single baseline models MLPR and SVR are not as good as the two coupled models in terms of R2 and NSE indicators, ranking in the last two. These results demonstrate that the coupled model is superior to the single reference model in extracting the key variables in the nonlinear data set and producing the prediction results accordingly.

RMSE, MAE, MAPE, and MSLE are effective indicators for evaluating the errors between model-predicted values and true values. They evaluate the deviation between predicted values and true observed values from different perspectives and can effectively mitigate the influence of outliers on model evaluation. Smaller values indicate closer proximity of predicted values to true values and better generalization performance of the model. Observing the performance of the coupled models (RF-SVR and RF-MLPR) compared to the corresponding baseline models (SVR and MLPR) in these four evaluation metrics at the LHK hydrological station, we see that RF-SVR reduces the RMSE, MAE, MAPE, and MSLE error indicators by 19.2%, 26.4%, 12.9%, and 23%, respectively, compared to SVR. Similar improvements are observed for RF-SVR compared to SVR at the JP, GD, and ET hydrological stations. Particularly notable is the 38% reduction in the MSLE indicator value of RF-SVR compared to SVR at the JP hydrological station. The coupled model RF-MLPR exhibits a similar pattern to MLPR in all four error evaluation indicators. At the LHK hydrological station, RF-MLPR reduces the RMSE, MAE, MAPE, and MSLE errors by 19.2%, 26.4%, 12.9%, and 37.4%, respectively, compared to MLPR. Similar reductions in the four error indicators are also observed at the JP, GD, and ET hydrological stations. At the ET hydrological station, RF-MLPR reduces the MAPE and MSLE errors by 54% and 42%, respectively, compared to MLPR. Through the comparison of the above prediction results, we can clearly observe that the coupled models perform significantly better than the baseline models on the datasets of the four different hydrological stations. This finding strongly demonstrates the effectiveness and superiority of the two coupled models constructed in this study for medium- to long-term runoff prediction.

By comprehensively comparing the above prediction results, it is evident that both the coupled models and single models exhibit good performance in terms of R-Square values (all above 0.6), demonstrating good nonlinear fitting capabilities. However, the prediction results of the coupled models are notably more outstanding in terms of R-Square values, further highlighting their superiority. Compared to single models, the coupled models show a significantly higher degree of closeness between predicted values and actual runoff observations, particularly evident in the performance of the R-Square indicator. Especially for hydrological stations with large downstream runoff volumes, the coupled models can significantly reduce prediction error values across all four error indicators compared to single models.

The above findings indicate that, relative to single models, coupled models can fully leverage the advantages of each module, thereby significantly improving the accuracy of medium- to long-term runoff prediction.

### 3.5 Evaluation of predictive results of two coupled intelligent models during the same prediction period

As illustrated in Fig 2 provided previously, substantial fluctuations are evident in the monthly runoff volumes across four representative hydrological stations within the YLRB. Remarkably, a discernible trend of escalating variability in runoff fluctuations is observed from upstream to downstream stations. Additionally, all four hydrological stations covered in the study exhibit noticeable fluctuations in runoff. The presence of such variability not only poses a significant challenge during the model prediction process but also serves as a rigorous test of the predictive performance of the model.

#### 3.5.1 Analysis of runoff prediction results for LHK hydrological station and JP hydrological station

**Fig 10:**
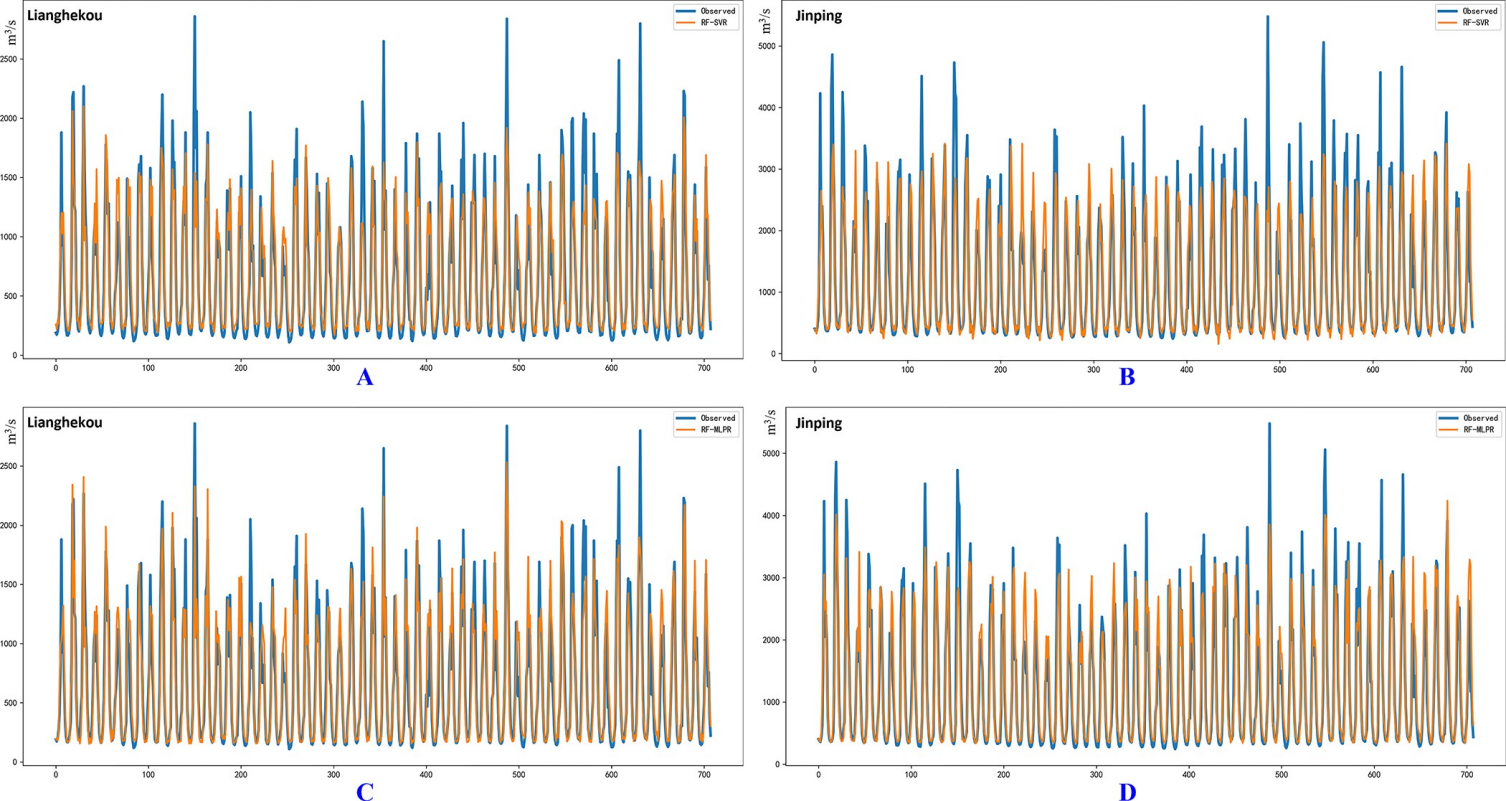
Predicted plots of runoff datasets for LHK and JP hydrological stations. Subplots A and C show the predicted results of the RF-SVR and RF-MLPR models at the LHK hydrological station, respectively. Subplots B and D depict the predicted results of the RF-SVR and RF-MLPR models at the JP hydrological station.

Fig 10 presents a visual comparison between the predicted runoff values from the RF-SVR and RF-MLPR models against the actual observed runoff data across the entire dataset for two hydrological stations: LHK and JP. The blue lines represent the actual observed runoff values, while the orange lines represent the predictions from the respective models. The figure highlights the predictive performance of both models across different temporal points, focusing particularly on the accuracy in capturing peak and low runoff events.

Subfigures A and B display the predictions of the RF-SVR model for the LHK (A) and JP (B) hydrological stations, respectively. It is evident from the visual comparison that the orange line (RF-SVR predictions) deviates from the blue line (observed values) at several points, particularly around the peaks and troughs. This deviation suggests that the RF-SVR model struggles to capture the extreme fluctuations in runoff, which is crucial for hydrological forecasting, especially in flood or drought scenarios. Despite this, the RF-SVR model provides a reasonable approximation of the overall trend, though with noticeable discrepancies in the magnitude of peak runoff values.

Subfigures C and D show the predictions from the RF-MLPR model for the LHK (C) and JP (D) hydrological stations. In comparison to Subfigures A and B, the orange line (RF-MLPR predictions) in Subfigures C and D aligns much more closely with the blue line (observed values). Specifically, the RF-MLPR model more accurately predicts both peak and low runoff values, demonstrating its superior ability to model the complex, nonlinear relationships in the runoff data. The RF-MLPR model’s predictions follow the observed runoff values more closely, particularly during periods of high variability, as reflected in the more synchronized peaks and troughs.

**Fig 11:**
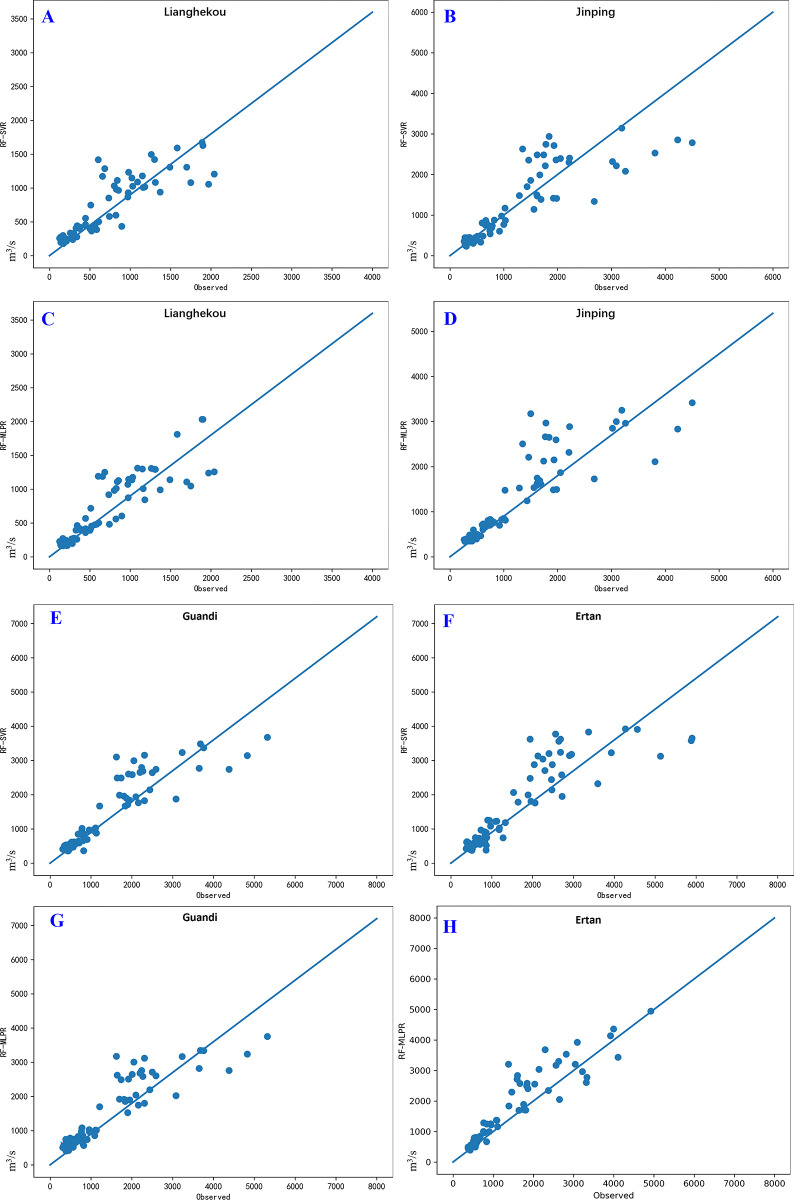
Regression plots of test data from LHK and JP hydrological stations. Subplots A and C display the predictive results of the RF-SVR model and RF-MLPR model, respectively, at the LHK hydrological station. Subplots B and D correspond to the predictive results of the RF-SVR model and RF-MLPR model at the JP hydrological station. Subplots E and G correspond to the predictive results of the RF-SVR and RF-MLPR models at the GD hydrological station, respectively. Subplots F and H correspond to the predictive results of the RF-SVR and RF-MLPR models at the ET hydrological station, respectively.

Fig 11 presents regression plots comparing the performance of the RF-SVR and RF-MLPR models for predicting runoff at four hydrological stations: HK, JP, GD and ET. The horizontal axis represents the actual observed runoff values at the respective stations, while the vertical axis represents the predicted runoff values. A perfect prediction would result in data points aligning closely along the diagonal straight line (y = x), indicating a high level of agreement between observed and predicted values.

Subfigures A and C display the results for the LHK station, comparing predictions from the RF-SVR (Subfigure A) and RF-MLPR (Subfigure C) models. The data points in Subfigure C (RF-MLPR) are more densely clustered around the diagonal line, indicating a higher consistency between the actual observed and predicted values compared to Subfigure A (RF-SVR). The RF-MLPR model demonstrates greater accuracy for this station. Similarly, Subfigures B and D compare the RF-SVR (Subfigure B) and RF-MLPR (Subfigure D) models for the JP station. As with the LHK station, Subfigure D shows a denser clustering of points along the diagonal, indicating that the RF-MLPR model provides better predictive performance than RF-SVR for this station as well.

Subfigures E and G present the results for the GD station, where Subfigure G (RF-MLPR) exhibits a tighter clustering of data points along the diagonal compared to Subfigure E (RF-SVR). This again suggests that the RF-MLPR model achieves higher predictive accuracy at the GD station. Finally, Subfigures F and H show predictions for the ET station. Subfigure H (RF-MLPR) displays a denser and more consistent alignment of points around the diagonal, indicating superior performance of the RF-MLPR model over the RF-SVR model (Subfigure F) for this station.

The comparison between RF-SVR and RF-MLPR in Fig 11 clearly demonstrates the superior predictive performance of the RF-MLPR model across all hydrological stations. The tighter clustering of data points around the diagonal in the RF-MLPR plots suggests that this model better captures the complex, nonlinear relationships inherent in the runoff data. This performance advantage can be attributed to the deep learning architecture of RF-MLPR, which enables it to generalize more effectively across varying datasets, particularly in high-dimensional and nonlinear contexts. In contrast, the RF-SVR model exhibits a more scattered pattern, reflecting its limitations in modeling such complexity. The consistent superiority of RF-MLPR at all stations underscores its potential as a more reliable and accurate model for medium- to long-term runoff forecasting, making it well-suited for practical applications in water resource management and environmental monitoring.

#### 3.5.2 Analysis of runoff prediction results for the GD hydrological station and ET hydrological station

**Fig 12:**
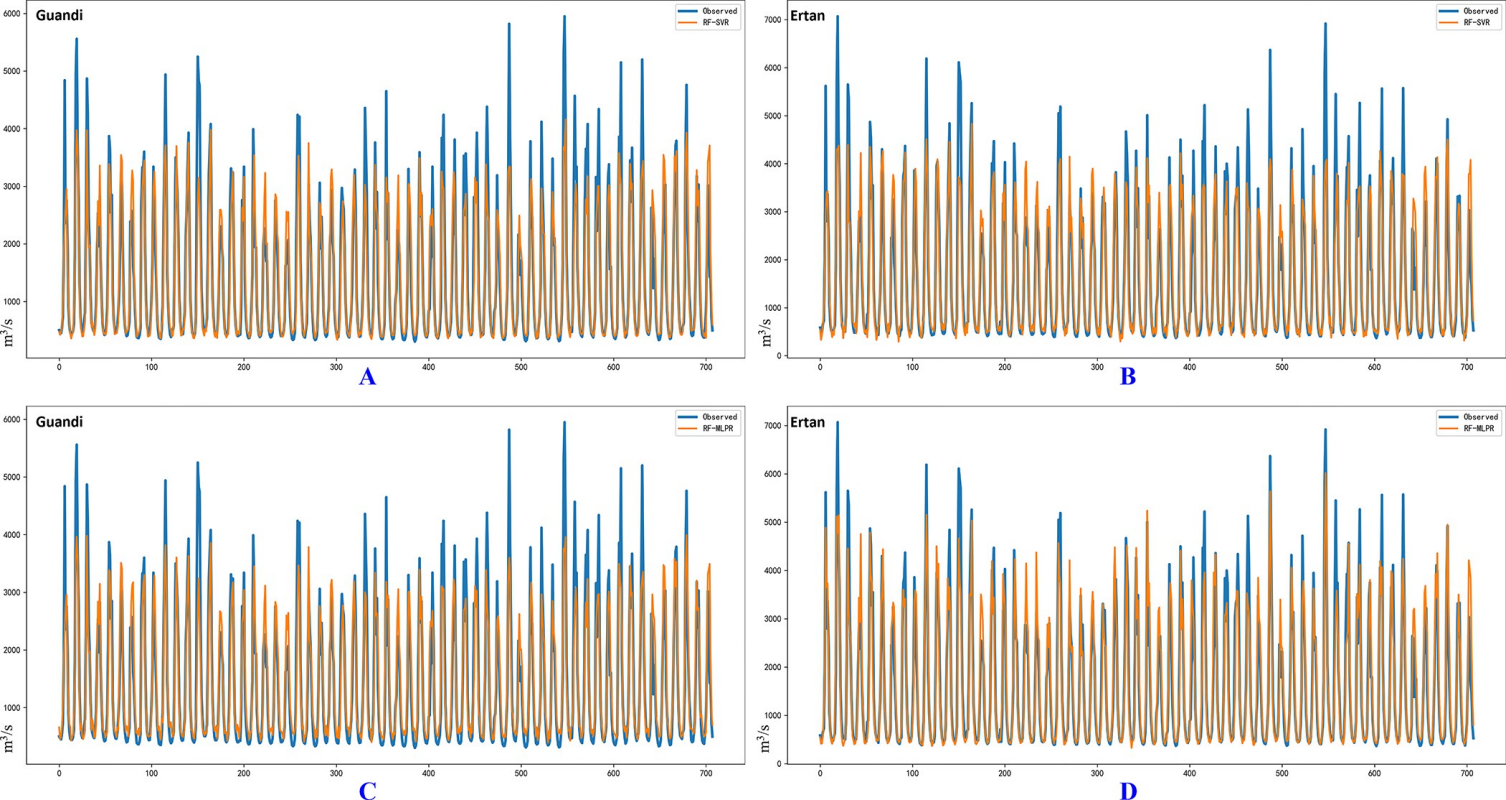
Predictive graphs of the RF-SVR and RF-MLPR models for the comprehensive dataset at the GD and JP hydrological sites. Subplots A and C depict the forecasted outcomes obtained using the RF-SVR and RF-MLPR models, respectively, at the LHK hydrological station. Subplots B and D correspond to the results of predictions made at the JP hydrological station using the RF-SVR and RF-MLPR models.

Fig 12 illustrates the monthly runoff forecast results for the GD and ET hydrological station datasets using the RF-SVR and RF-MLPR models. Similar to the Fig 10, the blue lines in the figure represent the actual observed runoff values for each hydrological station, while the orange lines represent the corresponding predictions from the coupled models.

Subfigures A and C of Fig 12 show the runoff prediction results for the GD hydrological station using the RF-SVR (A) and RF-MLPR (C) models. Both models exhibit similar trends in the predicted runoff curves, indicating that they have comparable generalization capabilities on the GD test dataset and can effectively capture the overall runoff pattern. However, upon closer examination of the peak points, the RF-MLPR model consistently outperforms the RF-SVR model, particularly in predicting the last peak in the sequence. The RF-MLPR model’s predictions align more closely with the observed values for both the second-to-last and fourth-to-last peaks, demonstrating its superior accuracy in capturing extreme runoff events. This advantage is especially evident during high runoff periods, where precise predictions are critical for flood forecasting and water resource management.

Subfigures B and D of Fig 12 present the results for the ET hydrological station, comparing the performance of the RF-SVR (B) and RF-MLPR (D) models. While both models effectively predict the overall runoff trend, it is evident that the RF-MLPR model provides more accurate predictions, particularly in terms of peak runoff values. The RF-MLPR model’s predictions align more closely with the actual observed runoff data, especially in the peak regions of the runoff process. The superior performance of the RF-MLPR model in capturing peak runoff points is critical, as accurate predictions of extreme values are essential for flood control and effective water resource planning. This improved accuracy is consistently observed throughout the prediction process, where the RF-MLPR model maintains closer adherence to the observed values compared to the RF-SVR model.

Fig 12 clearly demonstrates that while both RF-SVR and RF-MLPR models can capture the general trends in runoff prediction, RF-MLPR consistently exhibits superior performance, particularly in predicting peak runoff events. This distinction is especially significant for hydrological forecasting, where the accurate prediction of extreme values is crucial for effective flood control and water resource management. In both the GD and ET hydrological stations, the RF-MLPR model more closely aligns with the actual observed runoff values, particularly during peak runoff periods where the RF-SVR model shows greater deviations. This enhanced ability to model nonlinear and complex patterns inherent in runoff data underscores the robustness of the RF-MLPR model, especially in predicting high-magnitude runoff events, which are critical for practical applications.

Moreover, the RF-MLPR model’s consistent accuracy across different stations demonstrates its strong generalization capabilities, making it a reliable tool for various hydrological environments. The improved accuracy in predicting extreme values, such as peak runoff points, highlights the model’s potential for significantly enhancing decision-making in flood prevention and water resource planning. These capabilities allow for more precise and proactive interventions during periods of high runoff, showcasing the RF-MLPR model’s broad applicability and effectiveness in real-world scenarios where accurate hydrological forecasting is essential.

#### 3.5.3 Analysis of prediction results between two coupled models

**Fig 13:**
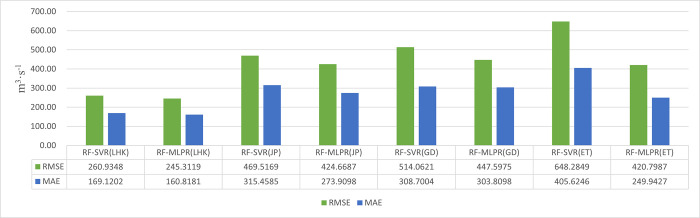
Evaluation values of RMSE and MAE for the prediction results of the RF-SVR and RF-MLPR models at 4 hydrological stations in the YLRB.

**Fig 14:**
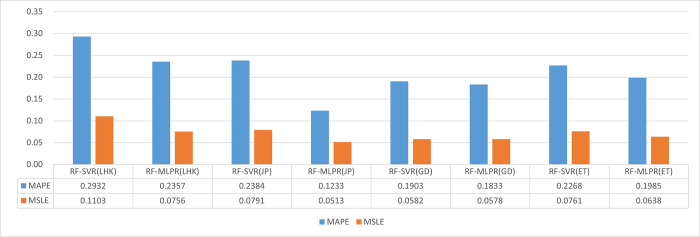
Evaluation values of MAPE and MSLE for the prediction results of the RF-SVR and RF-MLPR models at 4 hydrological stations in the YLRB.

In order to further investigate the performance of the two coupled models in predicting runoff, this section utilizes four error indicators to analyze their accuracy and effectiveness, followed by a performance comparison. Through Figs 13 and 14, we provide a detailed comparison of the performance of RF-SVR and RF-MLPR on the datasets of four different hydrological stations.

From the results presented in Fig 13, it is evident that the RF-MLPR model consistently outperforms the RF-SVR model across all hydrological stations in both RMSE and MAE metrics. This performance distinction is particularly notable at the ET hydrological station, where the RF-MLPR model shows a 35% reduction in RMSE and a 38% reduction in MAE compared to the RF-SVR model. Such a substantial improvement highlights the RF-MLPR model’s superior ability to capture the complex, nonlinear relationships present in the runoff data.

Additionally, a clear trend can be observed as we move from upstream (LHK) to downstream (ET). There is a gradual increase in both RMSE and MAE values for both models as we transition from the LHK station to the ET station. This increase in error values suggests that runoff prediction becomes more challenging at downstream stations, where the influence of anthropogenic factors—such as urbanization, agricultural activities, and infrastructure development—complicates the runoff dynamics. As hydrological stations move from sparsely populated upstream areas to more densely populated downstream regions, the factors affecting runoff become more diverse and harder to predict, contributing to the rise in error metrics.

The increasing RMSE and MAE values from upstream to downstream underscore the growing complexity of the runoff dynamics as anthropogenic factors come into play. The RF-MLPR model’s superior performance in reducing prediction errors across all stations suggests that it is better equipped to handle the nonlinearities and diverse factors influencing runoff. The model’s ability to maintain relatively lower error values at the downstream ET station is particularly notable, as this station is where runoff prediction is most challenging due to the increased human activities and environmental variability. These findings highlight the practical value of the RF-MLPR model in real-world hydrological forecasting, especially in regions where human intervention has a significant impact on runoff processes.

Fig 14 presents the MAPE and MSLE values for the RF-SVR and RF-MLPR models across four hydrological stations—LHK, JP, GD, and ET. The blue bars represent the MAPE values, while the orange bars represent the MSLE values for both models at each station.

The data clearly show that the RF-MLPR model consistently achieves lower prediction errors in both MAPE and MSLE metrics compared to the RF-SVR model across all four hydrological stations. This suggests that RF-MLPR demonstrates superior accuracy and generalization capability, making it more effective at handling the nonlinear, complex relationships in hydrological data.

At the JP station, the improvement in the RF-MLPR model’s performance is particularly striking, where the MAPE is reduced by 48% compared to the RF-SVR model. This significant reduction in MAPE at the JP station highlights RF-MLPR’s enhanced ability to minimize percentage errors, especially in challenging prediction scenarios where nonlinear variations in the runoff data are present.

Similar trends are observed at the LHK, GD, and ET stations, where the RF-MLPR model consistently shows lower MAPE and MSLE values than the RF-SVR model. For instance, at the LHK station, the RF-MLPR model achieves a MAPE of 0.2357, while the RF-SVR model records a higher value of 0.2932, representing a considerable improvement in prediction accuracy. Likewise, the RF-MLPR model shows superior performance in terms of the MSLE metric, with a reduction of 31% in MSLE at the LHK station compared to RF-SVR.

The consistent reduction in both MAPE and MSLE metrics for the RF-MLPR model across all stations indicates that this model is better equipped to capture the variability and complexity of the runoff data. The lower **MAPE** values highlight the relative accuracy of the models, with the RF-MLPR model delivering significantly fewer percentage errors, making it more reliable in practical applications where minimizing deviations from observed values is crucial. Similarly, the reduced MSLE values emphasize the RF-MLPR model’s ability to handle large errors more effectively, particularly in instances where the observed runoff values are small. This improvement is critical for enhancing the overall robustness of the model’s predictions.

The superior performance of the RF-MLPR model can be attributed to its deep learning architecture, which allows it to model complex, nonlinear relationships more effectively than the RF-SVR model, which struggles with the intricate aspects of runoff prediction. The substantial reduction in error values, particularly at the JP and LHK stations, demonstrates the RF-MLPR model’s capacity to provide more accurate forecasts, especially in scenarios where precise predictions are essential for decision-making, such as flood control and water resource management.

These results highlight the practical advantages of using the RF-MLPR model over RF-SVR for medium- to long-term runoff prediction. The model’s superior generalization capabilities, as evidenced by its lower error values across all stations, make it a more reliable tool for hydrological forecasting. The RF-MLPR model’s enhanced performance underscores its potential for providing more accurate forecasts in complex hydrological environments, further validating its applicability to diverse hydrological contexts where prediction accuracy is critical.

### 3.6 Comparing model performance: Using Taylor diagram analysis

**Fig 15:**
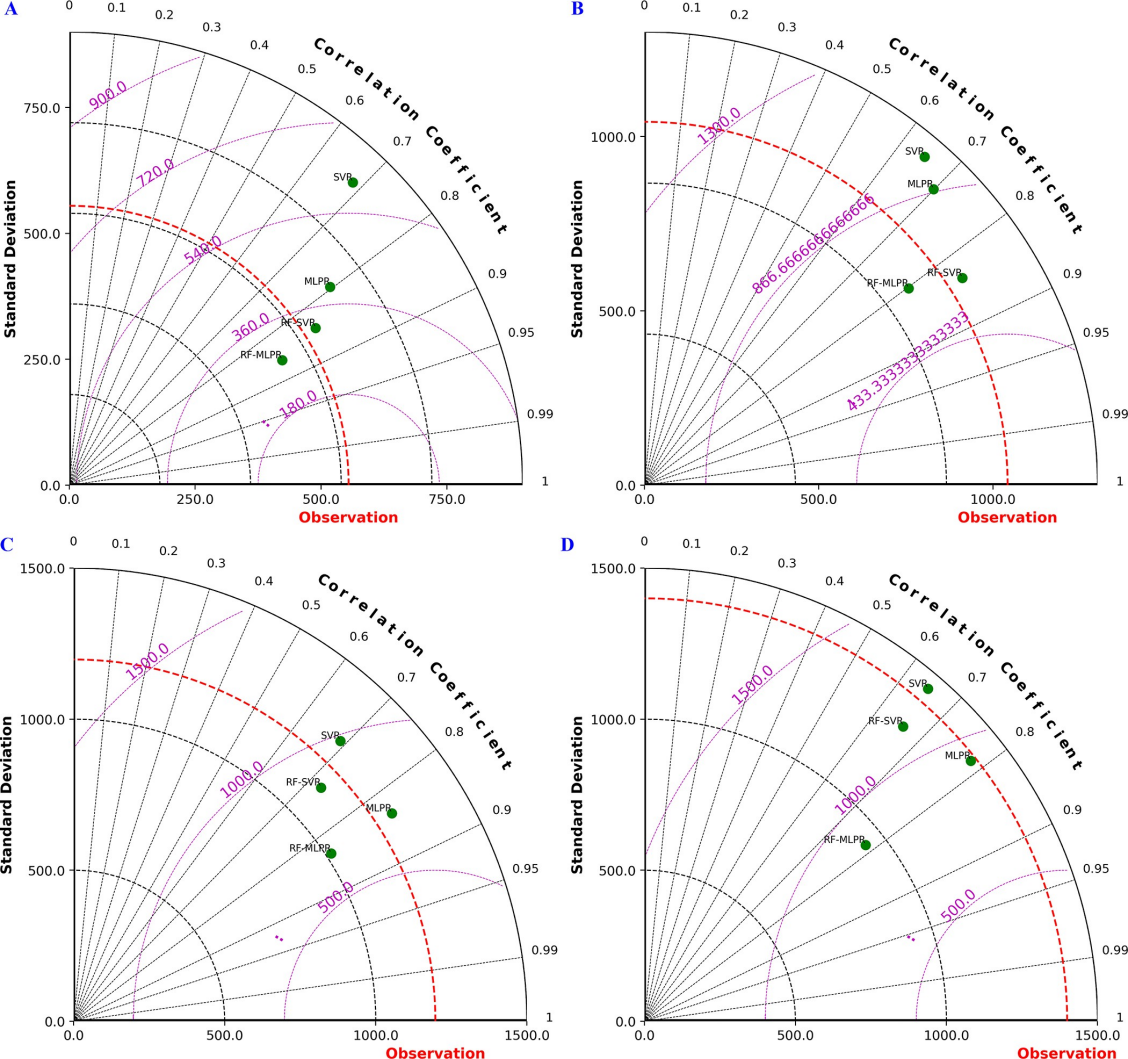
Taylor diagrams for each model for different station test periods. Subplots A, B, C, and D correspond to the Taylor diagrams of prediction results for the LHK, JP, GD, and ET hydrological stations, respectively.

To intuitively present the prediction performance of the single models and coupled models at the four hydrological stations, we utilized Taylor diagrams to conduct an in-depth analysis of the prediction results. Fig 15 showcases the Taylor diagram analysis of the prediction outcomes from different models during the testing phase, integrating three key evaluation metrics: Standard Deviation (SD), Root Mean Square Deviation (RMSD), and Correlation Coefficient (CC). In the diagram, the horizontal and vertical axes represent the RMSD and SD, respectively, while the radial axis denotes the correlation coefficient.

In the Taylor diagram, the RMSD is depicted as the radius of the concentric circles, with an RMSD of zero at the center. The closer a model’s point is to the origin, the better its prediction accuracy. Models with predictions near the observation point (marked in red) exhibit higher accuracy in capturing both the variability and the overall behavior of the runoff data. The RF-MLPR and RF-SVR models are positioned closer to the observation point compared to other models, indicating their superior performance in terms of prediction accuracy. This is particularly visible across all four subfigures (A, B, C, and D), where the green markers for RF-MLPR and RF-SVR consistently appear nearer to the origin, denoting lower RMSD and higher correlation with observed runoff data.

The Taylor diagram analysis in Fig 15 demonstrates the clear superiority of the RF-MLPR and RF-SVR models in predicting runoff data across all four hydrological stations. The proximity of the RF-MLPR and RF-SVR models to the observation point, which represents perfect agreement between predicted and observed values, indicates their ability to accurately capture both the variability (as measured by standard deviation) and the overall correlation with the observed data. Among these, the RF-MLPR model consistently outperforms the RF-SVR model, maintaining a closer alignment with the observation point, reflecting lower RMSD and higher correlation coefficients.

The diagrams further highlight the robustness and stability of the coupled models (RF-MLPR and RF-SVR) in comparison to single models. The consistent performance across all stations, demonstrated by the clustering of RF-MLPR points near the origin, underscores the model’s superior generalization capabilities in hydrological forecasting. This suggests that the coupled models are better equipped to handle the complex, nonlinear dynamics of runoff processes, offering reliable predictive performance even under varying hydrological conditions.

These findings have significant practical implications for medium- to long-term runoff forecasting. The superior accuracy and stability of the RF-MLPR model across different stations confirm its value as a reliable tool for critical applications such as flood prediction and water resource management. The model’s ability to consistently provide accurate predictions in diverse hydrological environments reinforces its potential to improve decision-making in scenarios that demand high predictive precision.

### 3.7 The impact of different prediction period on the predictive results of the coupled models

**Fig 16:**
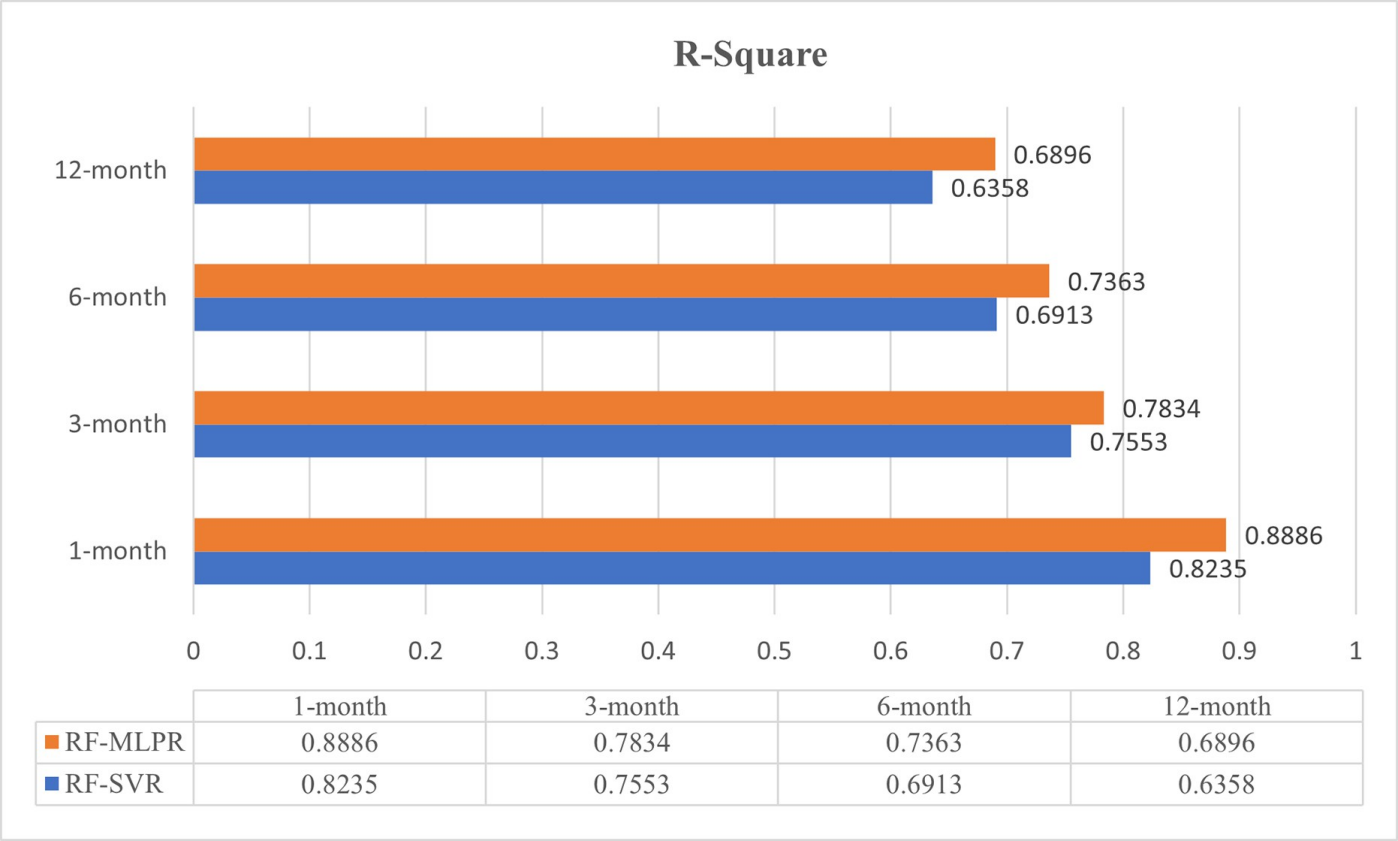
R-Square values of the two coupled models for different prediction period.

**Fig 17:**
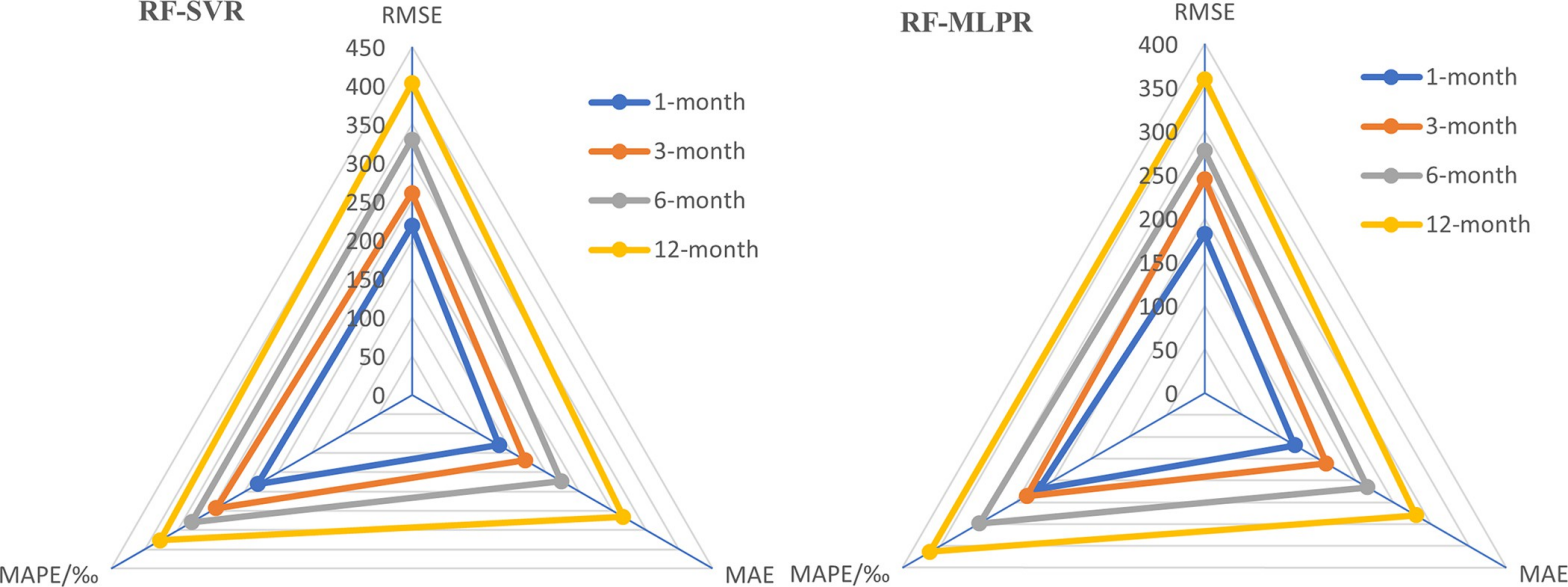
The runoff errors of RF-SVR and RF-MLPR at different prediction period.

The five metrics of runoff prediction results by the coupled model for four different prediction period are shown in **Table 6**. Due to space limitations, we will only analyze the Lianghekou hydrological station as an example here. To obtain intuitive results, histograms of R-Square values for different prediction period are presented in **Fig 16**. Additionally, radar charts of RMSE, MAPE, and MAE for different prediction period are illustrated in **Fig 17**.

**Table 6:** The evaluation metrics of the coupled model at the LHK hydrological station for different prediction period.

Predict Period	Coupled Model	*R-Square*	*RMSE/m* ^*3*^	*MAE/m* ^*3*^	*MAPE*	*MSLE*
**1-month**	**RF-SVR**	0.8235	218.6273	130.4683	0.2308	0.0829
	**RF-MLPR**	**0.8886**	**182.2365**	**119.5398**	**0.2196**	**0.0398**
**3-month**	**RF-SVR**	0.7553	260.9348	169.1202	0.2932	0.1103
	**RF-MLPR**	**0.7834**	**245.3119**	**160.8181**	**0.2357**	**0.0756**
**6-month**	**RF-SVR**	0.6913	330.3268	223.6081	0.3298	0.1568
	**RF-MLPR**	**0.7363**	**277.6832**	**215.8791**	**0.2983**	**0.1118**
**12-month**	**RF-SVR**	0.6358	403.3291	316.3892	0.3769	0.2597
	**RF-MLPR**	**0.6896**	**359.7863**	**280.0321**	**0.3638**	**0.1502**

Observing Fig 16, it can be seen that within prediction period of 1, 3, 6, and 12 months, both coupled models achieve relatively high prediction accuracy (R-Square values all exceeding 0.6), with the R-Square values of RF-MLPR significantly higher than those of RF-SVR. However, it is also noted that with the increase in prediction period, the R-Square values of both RF-SVR and RF-MLPR coupled models decrease significantly. In the RF-MLPR model, this is manifested by the R-Square value decreasing from 0.8886 at a prediction period of 1 month to 0.6896 at a prediction period of 12 months. Similarly, in the RF-SVR model, the R-Square value decreases from 0.8235 to 0.6358 over the same prediction period range. It is evident that an increase in prediction period results in a decrease in the prediction accuracy of the models.

To further quantitatively assess the effectiveness of the coupled models, this study compares the prediction error metrics of RMSE, MAE, and MAPE for both coupled models at different prediction period (Fig 17). Observing Fig 17, it is evident that all three error metrics increase as the prediction period of the two coupled models extends. In terms of MAE error metric, the RF-SVR coupled model exhibits an increase of 29.6%, 32.2%, and 42.4% across the four different prediction periods, while RF-MLPR shows increases of 34.5%, 34.2%, and 29.7% in MAE across the same prediction periods. Similar increasing trends are observed in the remaining error metrics, including RMSE and MAPE. The decrease in R-Square values and the increase in error metrics with longer prediction periods indicate a gradual decrease in the accuracy of model predictions over longer prediction periods.

## 4 Discussion

Accurate and reliable medium- to long-term runoff prediction is of significant importance for rational water resource planning and management, flood control and disaster reduction, maximizing the comprehensive benefits of reservoirs, and improving the ecological environment [1, 58]. The formation of runoff is influenced by numerous natural and human activities, making it a nonstationary, nonlinear, and complex process. Traditional approaches that solely consider the trend and information from individual runoff time series data or rely on statistical methods struggle to achieve high predictive accuracy, which is increasingly insufficient to meet the demands of current societal and industrial development. Recent research has found that traditional statistical series models, which only consider the trend and information from individual runoff time series data, are no longer sufficient to address the multifaceted factors influencing time series changes, nor can they meet the requirements for accuracy and robustness in hydrological forecasting, including precipitation and runoff prediction.

In light of this background demand, we incorporated, analyzed, and temporally and spatially aligned 88 datasets of atmospheric circulation indices. With the assistance of the Random Forest (RF) algorithm, we eliminated redundant and collinear variables and carefully selected key atmospheric circulation indices. We constructed two coupled intelligent models: the RF-SVR coupling model based on machine learning and the RF-MLPR coupling model based on deep learning. Instance verification at four main hydrological stations in the YLRB demonstrated that both coupling models effectively selected key atmospheric circulation indices significantly affecting runoff at the basin’s hydrological stations, resulting in more accurate runoff prediction results compared to the baseline models.

### 4.1 Typical atmospheric circulation indices affecting basin runoff

Observing the major atmospheric circulation indices identified by the two coupling models as having a significant impact on the runoff datasets at the four different hydrological stations, it becomes apparent that there is considerable overlap among the primary atmospheric circulation influencing factors at each station. This not only confirms the accurate identification by the coupling models of the fact that the four hydrological stations are spatially and temporally adjacent but also reflects that these selected atmospheric circulation indices are the most influential potential driving factors for runoff in the YLRB.

Upon observation, it is noted that the top four atmospheric circulation indices are the same across all four stations. They are: the Northern Hemisphere Polar Vortex Central Longitude Index, the East Asian Trough Intensity Index, the Asia Polar Vortex Intensity Index, and the Northern Hemisphere Polar Vortex Intensity Index. The following analysis delves deeper into these four atmospheric circulation indices from the perspective of how climate change induces changes in hydrological phenomena.

### 4.2 Northern Hemisphere Polar Vortex central longitude index

The Northern Hemisphere Polar Vortex is a large-scale low-pressure system located over the polar regions of the Northern Hemisphere, primarily composed of strong westerly winds that encircle the Arctic. It exerts a significant influence on global weather patterns and climate change. At the 500hPa level, the polar vortex typically manifests as a region of lower potential heights. Changes in the longitudinal position of the polar vortex have a considerable impact on the climate of mid-latitude regions. This is because it can alter atmospheric circulation patterns, particularly affecting the position and intensity of the westerly jet stream, thereby changing the activity paths of weather systems and influencing the pressure distribution in downstream areas. The YLRB is situated in the southeastern part of the Qinghai-Tibet Plateau. During winter, the westerly jet stream typically shifts southward, covering the Qinghai-Tibet Plateau and its southeastern regions. This geographical location makes the precipitation, runoff, temperature, and seasonal variations in the YLRB susceptible to influences from both the westerly jet stream and the monsoon climate system. Therefore, the longitudinal position of the Northern Hemisphere Polar Vortex center can modulate the monsoon climate system by affecting atmospheric circulation patterns and the position and intensity of the westerly jet stream, thereby exerting a significant influence on hydrological processes such as precipitation and runoff in the YLRB.

#### 4.2.1 East Asian Trough intensity index

The East Asian Trough is a critical weather system that plays an important role in the weather and climate variability of East Asia and the northwest Pacific region. When defining the "East Asian Trough Intensity Index," the characteristics of the trough within the 30°N-55°N and 110°E-170°E region at the 500hPa height field are considered. This index quantifies the intensity of the East Asian Trough by calculating the sum of potential heights at each point along the trough line, subtracting the maximum potential height, and adding the minimum potential height. This index reflects the activity characteristics of the East Asian Trough and subsequently influences downstream areas such as precipitation and runoff in the YLRB.

In terms of geographical location and influencing mechanisms, the YLRB is a typical high-mountain gorge area located in southwestern China, spanning the southeastern edge of the Qinghai-Tibet Plateau. This region is mainly influenced by the monsoon climate, with abundant precipitation often brought by the tropical monsoons from the Indian Ocean and the Pacific Ocean during the summer. As an important atmospheric circulation system, variations in the intensity and position of the East Asian Trough directly affect the intensity and distribution of the East Asian monsoon. When the intensity index is high, it typically indicates a deeper East Asian Trough, which can guide more cold air southward and interact with warm and moist airflows from the tropics and subtropics, increasing the probability of precipitation. When the East Asian Trough strengthens, the south wind ahead of the trough intensifies, facilitating the transportation of more warm and moist air to the YLRB, thereby increasing precipitation in the region. Precipitation is a primary factor in runoff formation. Therefore, changes in the East Asian Trough Intensity Index indirectly affect the runoff in the YLRB by influencing precipitation amounts.

#### 4.2.2 Asia Polar Vortex Intensity Index

The Asia Polar Vortex Intensity Index is defined by measuring the total air mass between the isopleths of the southern boundary of the polar vortex on the 500hPa pressure surface within the region of 60°E-150°E in the Northern Hemisphere. The YLRB is completely within the direct influence range of the polar vortex in the Asian region. The intensity and extent of the polar vortex affect the atmospheric circulation patterns in the Northern Hemisphere. A stronger polar vortex is usually accompanied by lower temperatures and a strong westerly jet stream, which can directly influence the atmospheric pressure systems and temperature distribution in the Asian region, thereby affecting the intensity and activity of the monsoon. Changes in the intensity of the polar vortex can indirectly alter the precipitation patterns in the YLRB by influencing atmospheric circulation and monsoon systems. When the intensity of the polar vortex strengthens, it may lead to more cold air moving southward, affecting the activity of the Asian monsoon, thereby increasing precipitation or altering precipitation patterns in the YLRB. Changes in the intensity and position of the polar vortex affect the moisture transport pathways between mid-high latitudes and low latitudes. Changes in intensity can lead to shifts in moisture transport pathways, affecting the humidity, precipitation, and runoff in the YLRB.

#### 4.2.3 Northern Hemisphere Polar Vortex Intensity Index

The Northern Hemisphere Polar Vortex Intensity Index, defined by measuring the total air mass between the isopleths of the southern boundary of the polar vortex on the 500hPa pressure surface, is an important indicator for assessing the overall intensity of the Northern Hemisphere polar vortex. As a major atmospheric circulation feature in the high latitudes of the Northern Hemisphere, the intensity and morphology of the polar vortex have significant impacts on the weather and climate in mid-latitudes and even low latitudes. The intensity of the polar vortex affects the temperature gradient between the polar regions and mid-latitudes, thereby influencing the atmospheric circulation patterns across the entire Northern Hemisphere. An increase in the intensity of the polar vortex generally results in the strengthening and confinement of polar cold air, along with the intensification of the westerly jet stream, which can alter the position and intensity of subtropical highs, impacting the weather and climate in low latitude regions. Changes in the intensity of the polar vortex can indirectly affect moisture transport pathways by altering atmospheric circulation patterns. For the YLRB, this implies that the strength of the winter and summer monsoons and the pathways of moist airflow will be affected, thereby exerting significant influences on the precipitation and runoff patterns in the region.

By focusing on monitoring and studying the variations represented by the aforementioned four atmospheric circulation indices, a better understanding and prediction of the hydrological and climatic conditions in the studied basin can be achieved, providing scientific basis for ecosystem protection, water resources management, and disaster prevention and mitigation in the region.

### 4.3 Advantages over traditional research

1) Given the issue of redundancy and collinearity among numerous introduced atmospheric circulation indices, we explored the use of the RF algorithm, an efficient and robust high-dimensional feature selection method. Under the conditions set in this study, only a few influential variables significantly affecting each basin site were retained, reducing model computation time and enhancing predictive accuracy [52, 59]. Traditional research often struggles to address collinearity issues, fails to effectively eliminate redundant variables, and accurately selects the feature variables that have a significant impact on the target basin hydrological site.

2) Combining Pearson correlation analysis, we investigated the lag period (approximately one month) of the influence of atmospheric circulation indices on the YLRB. This resolved the lag issue of atmospheric circulation indices affecting runoff over time, thereby improving prediction accuracy. However, traditional research mainly focuses on the direct application of atmospheric circulation indices and other external influencing factors [10, 60], often overlooking their temporal or spatial lag effects.

3) We found that as the observation stations extend from upstream to downstream, there is a gradual decrease in the accuracy of the model predictions. This trend may be related to the specific conditions of the YLRB. The upstream areas of the YLRB are mostly characterized by high mountains and deep gorges, with sparse human population. However, as we move downstream, human activities such as hydropower stations, large-scale constructions, and tourist attractions increase rapidly. The influence of these human activities has somewhat reduced the accuracy of the model predictions.

4) We investigated and compared the performance of the machine learning-based RF-SVR and deep learning-based RF-MLPR coupled models in runoff prediction. The optimally selected atmospheric circulation index features, enhanced by the RF component, are fed into the backend components of the coupled models in an end-to-end manner. Following multiple rounds of training, learning, and prediction steps, we achieved highly accurate predictive outcomes for the YLRB. Subsequently, we conducted comparative analyses to elucidate the advantages of the coupled models over single models in terms of prediction accuracy. Finally, we juxtaposed the prediction results of the RF-SVR and RF-MLPR models for four hydrological stations, with the LHK hydrological station as the representative, analyzing and discussing the runoff prediction effectiveness and variations in various evaluation metrics under different prediction period. Traditional methods often struggle to consider multiple variables influencing the target variable comprehensively [61], leading to lower predictive accuracy as they rely on single-model approaches and may fail to uncover hidden information embedded in high-dimensional variables.

In addition, we observed that after utilizing the RF algorithm to optimize the key variables influencing various hydrological sites, the RF-MLPR model consistently outperforms the RF-SVR model in terms of prediction results for both upstream and downstream sites within the basin. In other words, the deep learning-based RF-MLPR model demonstrates higher accuracy and generalization performance than the machine learning-based RF-SVR model. This is consistent with the superior capability of deep neural networks in nonlinear modeling and aligns with previous research [62–64] conclusions. Generally, when extracting latent information from nonstationary, nonlinear data, machine learning algorithms often require intricate and complex manual feature engineering to enhance model accuracy. However, the deep learning-based RF-MLPR model can automatically extract latent information hidden in complex data without human intervention. It then applies this information to the training and inference stages, resulting in accurate, stable, and well-generalized models, along with highly accurate predictive results. Figs 10–14 present the evaluation metric charts of the RF-MLPR and RF-SVR models for the four hydrological stations in the YLRB. Clearly, the evaluation metrics of the prediction results show that the RF-MLPR model consistently outperforms the RF-SVR model, whether at upstream or downstream sites. This indicates that the model based on deep learning possesses consistent stability across all sites and is capable of digging deep into latent information within the data to enhance prediction accuracy. Thus, both of the constructed coupled algorithm models in this study are capable of accurately and stably predicting medium- to long-term runoff volume. In particular, the RF-MLPR model exhibits higher accuracy and stability when predicting nonstationary, nonlinear data. The coupled runoff prediction intelligent models and methodologies proposed in this study can provide valuable references for other predictions in the hydrological field, environmental sciences, meteorological forecasts, and climate predictions.

### 4.4 Limitations and prospects

The research outcomes undoubtedly provide decision-making references for hydrological, meteorological, and climate predictions not only for the YLRB but also for other basins. Additionally, the RF-MLPR and RF-SVR models established in this study have practical significance and can be flexibly adjusted based on regional and data-specific parameters. However, there are still some limitations, which can be summarized into two parts:

1. To achieve more accurate prediction outcomes, both the machine learning-based RF-SVR model and the deep learning-based RF-MLPR model were employed. However, these models have numerous internal parameters and involve complex computational processes, making it challenging to provide easily interpretable explanations for the model predictions. This to some extent limits their application in certain critical domains.
2. Another aspect involves the applicability of the proposed method to other river basins. Although we have examined its competitive performance in the YLRB, the distinct characteristics of different basins, such as geographical features, vegetation and ecosystems, human activities, and land use, inevitably affect the accuracy and efficiency of the model.

Future work will focus on developing solutions that combine data-driven and algorithmic models with interpretability to address this challenge.

## 5 Conclusions

To address the issue of accuracy in medium- to long-term runoff forecasting, this study integrates the characteristics of hydrological and meteorological data to propose and construct two coupled intelligent models with feature selection capabilities: the RF-SVR model based on machine learning and the RF-MLPR model based on deep learning. Through in-depth comparative analysis of observed runoff data from four representative hydrological stations in the Yalong River Basin (YLRB), the results indicate that, compared to various single machine learning models, the constructed coupled models significantly improve prediction accuracy across multiple forecast periods. Moreover, these models effectively eliminate collinearity and redundant variable issues by leveraging the spatiotemporal correlation of the data, thereby enhancing the scientific rigor and objectivity of the prediction models. Notable conclusions are as follows:

1. Model prediction accuracy decreases at the downstream hydrological stations, where human activities have a greater influence. Upstream, natural convergence processes dominate, while downstream, factors such as hydropower, irrigation, and construction reduce predictive accuracy. For example, at the LHK hydrological station in the upper reaches of the Yalong River Basin, the RF-MLPR model’s R^2^ value was 2.4 percentage points higher than that at the ET hydrological station downstream. Similarly, in terms of the NSE metric, the model’s performance at LHK exceeded that at the ET station by 2.2 percentage points.
2. The study found a one-month lag in the impact of atmospheric circulation indices on runoff at upstream and downstream stations. Understanding this lag helps optimize water resource allocation and informs targeted flood control measures, maximizing the benefits of basin reservoirs.
3. Introducing atmospheric circulation indices as teleconnection factors addresses the limitations of relying solely on runoff data from hydrological stations. These indices provide more objective and natural factors for prediction, enhancing the model’s accuracy and reliability by reducing the influence of human intervention.
4. The coupled models efficiently handle multidimensional feature variables, automatically extracting those most relevant to the target variable. This resolves the issue of low effective data density in current hydrology data, improving prediction outcomes.
5. The two coupled intelligent models, RF-SVR and RF-MLPR, are suitable for medium- to long-term runoff prediction, achieving high predictive accuracy. In the YLRB, these models outperformed baseline models at four key hydrological stations, with RF-MLPR showing consistently superior performance across multiple metrics, demonstrating its deep learning-based ability to extract more comprehensive latent information. At the four hydrological monitoring stations from upstream to downstream, the RF-MLPR model achieved increases of 3.7%, 6.5%, 0.4%, and 0.2% in the NSE metric compared to the RF-SVR model, respectively.
6. The coupled models demonstrate superior accuracy compared to single baseline models across various hydrological stations and prediction periods. Specifically, the RF-SVR model improved prediction accuracy by up to 18.2% compared to the single baseline SVR model, while the RF-MLPR model achieved up to a 16.2% improvement over its respective baseline model. Their flexibility in parameterization makes them highly applicable to various regions and data conditions, providing a useful reference for multi-source data fusion in hydrology, environmental sciences, meteorology, and climate prediction.

This study significantly contributes to the existing body of research on medium- to long-term runoff forecasting by demonstrating the effectiveness of integrating teleconnection indices with advanced machine learning and deep learning models. By developing and validating the RF-SVR and RF-MLPR coupled models, the research addresses key limitations in traditional hydrological prediction models, particularly in managing high-dimensional, nonlinear data, and incorporating atmospheric circulation factors. The findings underline the importance of combining spatiotemporal data correlation and feature selection to improve predictive accuracy, especially in complex environments influenced by both natural and anthropogenic factors. Furthermore, the superior performance of the RF-MLPR model highlights the potential of deep learning techniques in hydrological forecasting, opening avenues for future studies to explore deeper architectures and multi-source data integration. Practically, the models presented in this study offer valuable tools for water resource management, flood prediction, and environmental monitoring, with the flexibility to adapt to various regions and climate conditions. The integration of atmospheric indices not only enhances the accuracy and reliability of predictions but also provides actionable insights for policy-makers and engineers involved in flood control and infrastructure planning, making the findings highly relevant for both theoretical advancements and real-world applications.
